# Supplementary material for: Annelid adult cell type diversity and their pluripotent cellular origins
Source: Nat Commun. 2024 Apr 12;15:3194. doi: 10.1038/s41467-024-47401-6 (PMC11014941; doi:10.1038/s41467-024-47401-6)
Supplement: Supplementary file 1 — Supplementary Information [file 41467_2024_47401_MOESM1_ESM.pdf]

Supplementary Information

Annelid adult cell type diversity and their pluripotent cellular origins

Patricia Álvarez-Campos [1, 2, *, †], Helena García-Castro [1, §, †], Elena Emili [1], Alberto Pérez-Posada [1, §], Irene del Olmo [2], Sophie Peron [1, §], David A. Salamanca-Díaz [1, §], Vincent Mason [1], Bria Metzger [3, 4], Alexandra E. Bely [5], Nathan J. Kenny [1, 6], B. Duygu Özpolat [3, 4, *] and Jordi Solana [1, §, *]

[1] Department of Biological and Medical Sciences, Oxford Brookes University, Oxford, UK
[2] Centro de Investigación en Biodiversidad y Cambio Global (CIBC-UAM) & Departamento de Biología (Zoología), Facultad de Ciencias, Universidad Autónoma de Madrid, Madrid, Spain
[3] Marine Biological Laboratory, 7 MBL Street, Woods Hole, MA, USA, 05432
[4] Department of Biology, Washington University in St. Louis. 1 Brookings Dr. Saint Louis, MO, USA, 63130
[5] Department of Biology, University of Maryland, College Park, MD 20742, USA
[6] Department of Biochemistry, University of Otago, P.O. Box 56, Dunedin, Aotearoa New Zealand

[*] Corresponding authors
[†] Equally contributing authors

[§] Present address: Living Systems Institute, School of Biosciences, Faculty of Health and Life Sciences, University of Exeter, Exeter, UK

Contents:

Supplementary Note 1

Supplementary Note 2

Supplementary Note 3

Figure Supplementary 1

Figure Supplementary 2

Figure Supplementary 3

Figure Supplementary 4

Figure Supplementary 5

Figure Supplementary 6

Figure Supplementary 7

Figure Supplementary 8

Figure Supplementary 9

Figure Supplementary 10

Supplementary References

Supplementary Note 1: Details of the single cell analysis.

We first obtained a new transcriptome from adult *Pristina* individuals (mixed stages, mRNA) using Iso-Seq on the PacBio Sequel II platform. From 1,546,939 CCS reads, we recovered 367,025 reads spanning 111,961 full transcripts, with a mean length of 1,664 bp (full transcript N50: 3796 bp). This data was combined with previously published transcriptomic resources [1] using EvidentialGene [2], resulting in a final non-redundant transcriptomic resource containing 37,263 transcripts and 96.3% of the metazoan BUSCO cassette. We annotated these transcripts using eggNOG [3] against the metazoan database and performing Diamond Blast [4] against a local version of the nr database (Supplementary Data 1).

We obtained cell dissociations of adult mixed populations of *Pristina* containing intact organisms in all fissioning stages (Figure 1A). We used ACME dissociation, a recently developed protocol that fixes the cells early during the dissociation process [5]. ACME produces cell suspensions that are fixed and permeabilised, and are therefore ideal for performing SPLiT-seq [6] or other single cell transcriptomic methods based on combinatorial barcoding. These methods tend to give less gene and UMI content per cell, but are in turn cost effective and allow obtaining tens of thousands of cell profiles at a low cost, maximising the detection of rarer cell types. We performed three independent SPLiT-seq experiments (Figure 1A) and sequenced them using the Illumina NovaSeq 6000 platform at 2x 150 bp read length. We processed these reads with our SPLiT-seq pipeline [5,6] and obtained a total of 80,387 cell profiles above 50 genes per cell. We aimed at identifying doublets using Scrublet [7] and Solo [8], obtaining 2,870 and 2,554 cell barcodes respectively (Supplementary Figure 1A). The overlap of both methods was 458 cells, a significant ($p = 0.006$, above expected $p = 0.001$) but moderate result that indicates that doublet identification tools have a low level of agreement on such a complex dataset. We then examined whether doublets have a large influence on the overall quality of the data, for instance by creating cell clusters dominated by doublets. We processed the dataset containing doublets and performed Leiden clustering in 4 different resolutions (Supplementary Figure 1B) ranging from 46 to 88 clusters. This revealed that even at high clustering resolutions there are no clusters dominated by Scrublet and/or Solo doublets (Supplementary Figure 1C), showing that Scrublet and Solo-detected doublets are not a major source of clustering formation.

We removed both doublet lists from the dataset, eliminating 4,966 cells (6.1%), a value conservatively above the ~3% doublet expectation of our SPLiT-seq experiments. We explored the preprocessing parameter space with this 75,421-cell dataset, revealing that most conditions generated 40-60 reproducible clusters at resolution 1, but that a large number of Principal Components (PCs) and Highly Variable Genes (HVG) were needed to capture the full structure of the dataset (Supplementary Data 2). We then processed the dataset eliminating the top expressed gene (PrileiEVm0194901t1), since this made up about 60% of our UMI counts, likely a product of SPLiT-seq's library amplification

process. We further eliminated cells above 700 genes and 900 raw UMI counts as likely doublets, obtaining a final dataset of 75,218 cells. We processed this dataset with 18,000 HVGsm, 45 neighbours and 105 PCs, obtaining a mean of 110 genes per cell and 397 UMI counts per cell (Supplementary Figure 2AB). Despite this relatively low gene and UMI count content, cell clustering with the Leiden algorithm (resolution 1.5) allowed us to robustly identify 60 cell clusters (Figure 1B, Supplementary Figure 2C-D, Supplementary Figure 3A) that are reproducible across parameter conditions (Supplementary Data 2), and have highly specific markers (Figure 1C, Supplementary Figure 2E). We calculated marker genes for each cluster using the Wilcoxon and the Logistic Regression methods (Supplementary Data 3 and 4), which showed a high but not complete overlap. In some clusters, one of the two methods performed better than the other, but no method performed best in all clusters (Supplementary Figure 2F). We report the overlapping markers (Supplementary Data 5). We noticed that some small clusters (46, 47, 48, 50, 51, 52, 53, 54, 56, 57, 58, ranging from 174 to 41 cells, 0.2% and 0.05% of the dataset respectively) shared the same UMAP space as clusters 1 and 2 (Supplementary Figure 3B) and expressed genes characteristic of these cell populations. These small clusters either represent subsets of the clusters 1 and 2 population or leftover doublets unidentified by Scrublet or Solo, and were flagged as "unannotated". The total amount of unannotated cells is 1,048 (1.39% of the total dataset), a very small number that is within the order of magnitude of expected doublets (~3%).

We then used PAGA [9] to reconstruct differentiation trajectories. When we performed PAGA on the full dataset, small unannotated clusters connected other groups of clusters with unrelated markers (Supplementary Figure 4), reinforcing the idea that they could represent doublets. We then performed PAGA using only annotated clusters (Figure 1D). This lineage reconstruction allowed us to classify the broad cell types (Figure 1E). We also performed a co-occurrence analysis of cell type clusters [10], using the gene expression data of highly variable genes, summed at the cell cluster level. This analysis broadly confirmed our cluster groups (Supplementary Figure 5).

Supplementary Note 2: Naming and grouping of the clusters.

To characterise the cell types represented by each of our cell clusters we analysed and discussed the markers of each cluster, their Diamond BLAST annotations, and put these into the context of the available annelid and single cell literature, together with our own data. Broad groups of cell types were obtained by leveraging the information of the PAGA analysis and the co-occurrence matrix.

***Piwi*+ cells**: We found that the expression of *piwi-1* concentrated in clusters 1 and 2, but also in cluster 8. A number of unannotated clusters were mixed with clusters 1 and 2 in the UMAP space and also expressed *piwi-1*.

Epidermis: We identified epidermal cells based on the analysis of the expression of the markers PrileiEVm008309t1 and PrileiEVm008287t1, encoding intermediate filament proteins.

Gut: We found this group of cell clusters connected to cluster 16, suggesting that cluster 16 contains gut progenitors. We identified and named the different regions of the *Pristina leidyi* gut based on the analysis of the expression of the markers PrileiEVm010132t1 (unannotated transcript), PrileiEVm010941t1 (unannotated transcript), PrileiEVm000199t1 (encoding a rootletin protein), PrileiEVm017310t1 (unannotated transcript), PrileiEVm019805t1 (unannotated transcript), PrileiEVm005677t1 (encoding a long-chain-fatty-acid-CoA ligase protein), PrileiEVm021761t1 (unannotated transcript), PrileiEVm001383t1 (encoding a von Willebrand factor D and EGF domain-containing protein), PrileiEVm026965t1 (unannotated transcript) and PrileiEVm020317t1 (unannotated transcript). We found that Cluster 38 was only connected to the group of gut cell clusters below the threshold and we named it after the marker PrileiEVm022227t1 (encoding a caveolin protein), but kept it in the broad gut group.

Muscle: We found that clusters 5, 6, 27 and 30 highly expressed components of the muscle sarcomere, including myosin heavy chain (PrileiEVm000300t1), myosin light chain (PrileiEVm019310t1, PrileiEVm025675t1, PrileiEVm020932t1), tropomyosin (PrileiEVm016172t1, PrileiEVm015909t1), troponin (PrileiEVm014978t1, PrileiEVm014978t1, PrileiEVm018738t1) and titin (PrileiEVm000447t1). Cluster 27 contains cells that are mixed with Clusters 8, 1 and 2, is connected by PAGA analysis to cluster 8, and likely represents a progenitor state.

Neurons: We identified the neuronal populations based on the expression of synaptotagmin (PrileiEVm012030t1) and other neuronal specific transcripts such as PrileiEVm000558t1, which *in-situ* expression marks the Ventral Nerve Cord (VNC). These markers were highly expressed in cluster 3, 40, 41, 42, 45, 49 and 59. Cluster 49 also expressed a homolog of arrestin protein-domain (PrileiEVm014226t1) suggesting a possible identity for this cluster as photoreceptor neurons.

***Globin*+ cells**: We found that clusters 4 and 33 highly expressed extracellular globins, including PrileiEVm020672t1, PrileiEVm018061t1, PrileiEVm020599t1, PrileiEVm020147t1.

***Polycystin* cells**: We named this cluster group based on the expression of *polycystin*-2 (PrileiEVm005033t1) and *polycystin*-1 (PrileiEVm004079t1) homologue genes.

Eleocytes: We identified this cluster based on the expression of a vitellogenin homologue (PrileiEVm000002t1) which was also expressed in *globin*+ cells. The 3 clusters interconnected in the PAGA graph were named after markers *mmp24* (PrileiEVm009033t1), *fucolectin* (PrileiEVm007557t1) and *pgrn* (PrileiEVm018088t1).

Chaetal sacs: We identified the chaetal sacs based on the expression of PrileiEVm000939t1 (encoding a kunitz-type protease inhibitor protein), PrileiEVm007502t1 (encoding an intermediate filament protein) and PrileiEVm000573t1 (encoding a chitin synthase protein).

***Lipoxygenase*+ cells**: We named this cluster based on the expression of several *lipoxygenase* transcripts, including PrileiEVm000278t1, PrileiEVm008087t1 and PrileiEVm008285t1.

***Vigilin*+ cells**: We named this cluster after the expression of the RNA binding protein *vigilin* (PrileiEVm001339t1).

***Lumbrokinase*+ cells**: We found that cluster 26 highly expressed several homologues of the lumbrokinase enzymes, including PrileiEVm016387t1, PrileiEVm016330t1, PrileiEVm016446t1 and PrileiEVm016633t1.

Carbohydrate metabolic cells: We named this cluster based on the expression of several transcripts annotated as enzymes involved in carbohydrate metabolism, including a fructose-bisphosphate aldolase (PrileiEVm013994t1), a phosphoenolpyruvate carboxykinase (PrileiEVm026130t1), a malate dehydrogenase (PrileiEVm014911t1), as well as mitochondrial enzymes such as a glutamate dehydrogenase (PrileiEVm008904t1), a pyruvate carboxylase (PrileiEVm001525t1), and a mitochondrial malate dehydrogenase (PrileiEVm026983t1).

Secretory: We proposed cluster 34 and 44 as secretory clusters based on the expression of a homolog of the conotoxin protein (PrileiEVm010163t1). The *in-situ* for this transcript marks a cluster of large cells segmentally repeating on the ventral side, after each chaetae bundle but not overlapping with the chaetae themselves. Also, some single cells in the posterior growth zone seem to be marked. These clusters also appear to express the synaptotagmin transcript (PrileiEVm012030t1) suggesting a possible neuro-secretory function.

***Arginase*+ cells**: We named this cluster group based on the expression of an arginase homologue, PrileiEVm015527t1

***Ldlrr+* cells**: We named this cluster group based on the expression of a low-density lipoprotein receptor homologue, PrileiEVm010669t1.

Metanephridia: We identified metanephridia cells based on the analysis of expression of the marker PrileiEVm002621t1.

Supplementary Note 3: Notes on the WGCNA graph analysis.

General rationale and TOM values:

When generating a coexpression network using WGCNA [11], we sought to investigate whether these gene-gene correlations exhibited a graph behaviour. For this we used the TOM adjacency matrix as an entry point to generate a graph where nodes are genes and edges are TOM connection values. We first explored the behaviour of the resulting graph by defining a set of increasing threshold values to iterate the generation and visualisation of a sub-graph. This code and the respective graph layouts can be found in our GitHub repository. Upon visual inspection of these graphs, we observed a shift in graph structure when removing edges below 0.35.

The majority of the WGCNA modules that we detected exhibit a very specific expression pattern in unique cell types. Thus, we wondered if graph analysis could prove useful to detect subtle patterns of gene coexpression and gene co-regulation in different cell types. If this were the case, the co-expression graph would exhibit connections between genes from the same modules more preferentially, which would correspond to connected components (CC), but also connections between genes from different modules to a lesser extent, which we call cross-connections. In order to determine the presence of connected components in the graph; i.e. the connections between genes from the same or related modules, we generated a sub-graph using a cutoff value of TOM interactions >0.35 (Figure 4E), using the Fruchterman-Reingold layout to plot the genes in the network. Of the 40 modules detected in WGCNA, 38 pass this threshold and are present in this graph. There are two modules (module_neurons3 and module_neurons5) for which all genes are connected by TOM scores below 0.35, and therefore do not appear in the graph (Figure 4E). We observed a high agreement between the connected components of this subgraph and the module assignment provided by WGCNA (Supplementary Figure 9A,B).

We argue that exploration of the presence of connected components in the structure of the graph is a prerequisite for the study of cross-connections, as it would not make sense to seek connections between modules if these were not grouped in connected components and the whole network was fuzzy. To discern if different modules are connected, i.e. to detect and explore module cross-connections, we subsetted the graph using a lax threshold (> 0.2)(Figure 4F).

On Transcription Factor (TF) connectivity and TF Centrality:

To ascertain the biological relevance of TF centrality, we explored TF centrality in relation to another important WGCNA metric called module connectivity. Based on Langfelder and Horvath [11,12], the metric of “connectivity to module” kME(x,y) is defined as the correlation of the gene expression of a given gene ‘x’ with the weighted average expression of all genes in a given module ‘y’. We reasoned

this definition could be extended in the case of TF genes as the likelihood/potential of a given TF gene to play a role in the regulation of the expression of the genes in a module. This definition goes along the same lines of current standard methods to infer the regulatory capacity of a TF based on gene expression [13-16].

We reasoned that, if a graph is constructed where genes are connected based on WGCNA coexpression values (such as in Fig. 4E,F,G), a TF ‘x’ connected to many genes of a given module ‘y’ should exhibit a high centrality C(x,y) as well as a high connectivity kME(x,y) value. Therefore, if there is such an agreement between the two metrics, we argue that centrality in a graph might also prove useful to identify potential regulatory TF candidates.

Since TF centrality is calculated independent of kME, we explored the relationship between these two metrics. We observed that TF genes that pass the Connected Component analysis (i.e. those present in the graph of Figure 4E) and thus have a centrality value calculated, show an overall higher connectivity (Supp. Fig 9C,D). This association between centrality and connectivity is intrinsic to each gene module, as every gene module sub-graph has distinct properties. Thus, high correlation values between kME and centrality only emerge when inspecting the relationship between these metrics for each module individually (Supplementary Figure 9E,F).

Provided most of the gene modules we found exhibit a strong, cell type-specific gene expression, we argue that it is possible to find potential regulatory TFs of cell type identity and function using both TF connectivity and TF centrality.

Figure S1

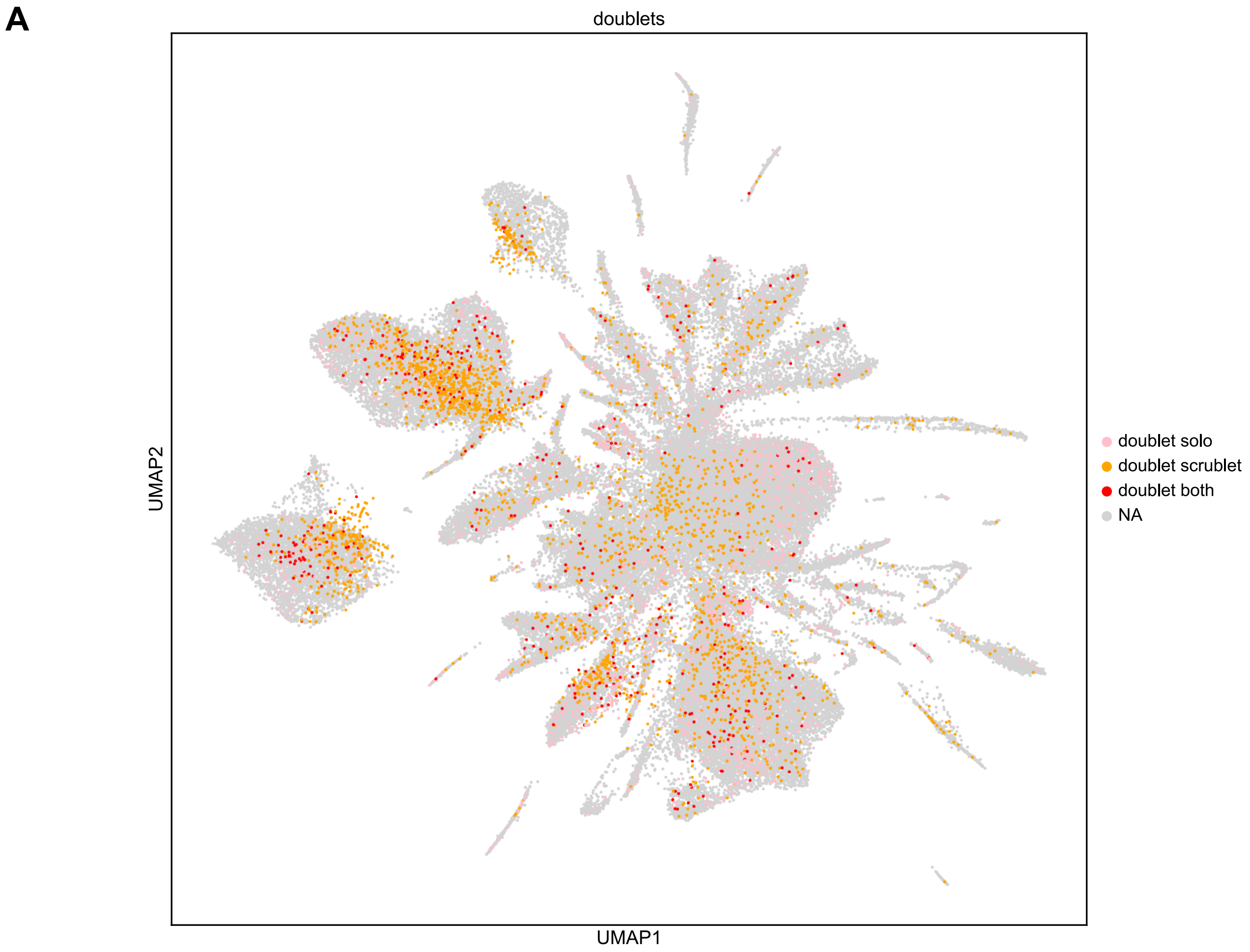
A
doublets
UMAP2
UMAP1
doublet solo
doublet scrublet
doublet both
NA

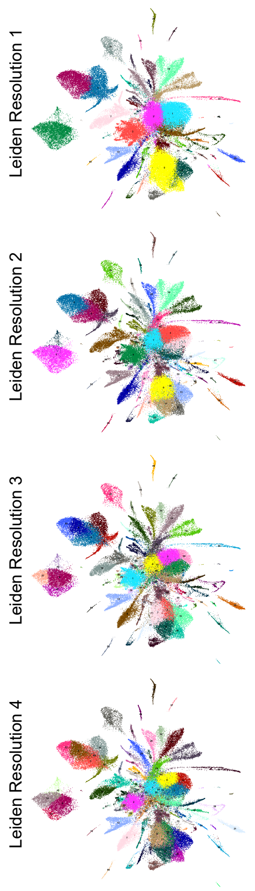
B
Leiden Resolution 1
Leiden Resolution 2
Leiden Resolution 3
Leiden Resolution 4

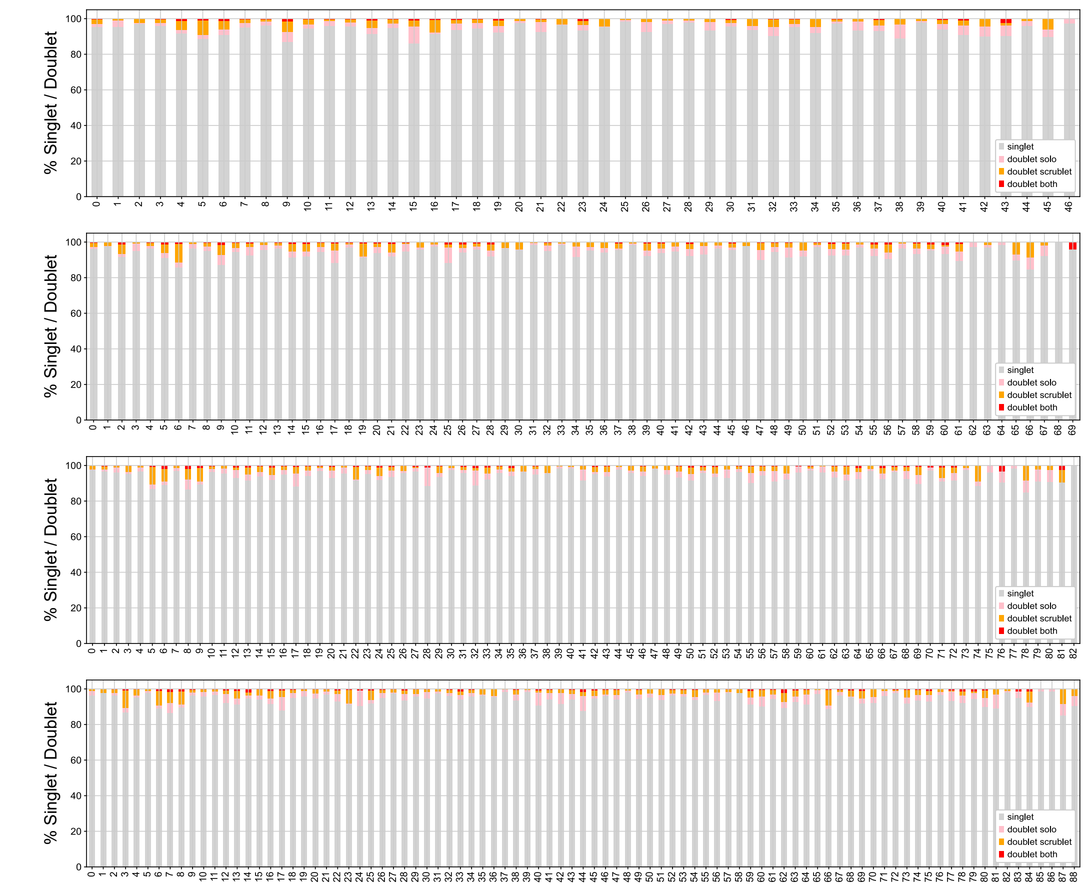
C
% Singlet / Doublet
singlet
doublet solo
doublet scrublet
doublet both

Figure Supplementary 1: Doublet identification with Solo and Scrublet.

A) UMAP visualisation of the 80,387 cells prior to doublet classification. Cells classified as doublets by Solo and Scrublet are highlighted in pink and orange respectively, and cells classified as doublets by both are highlighted in red.

B) UMAP visualisation of the 80,387 cell dataset after clustering in 4 different resolutions.

C) Percentage of cell classified as doublets by Solo, Scrublet and both methods in each cell cluster across the 4 different resolutions

Figure S2

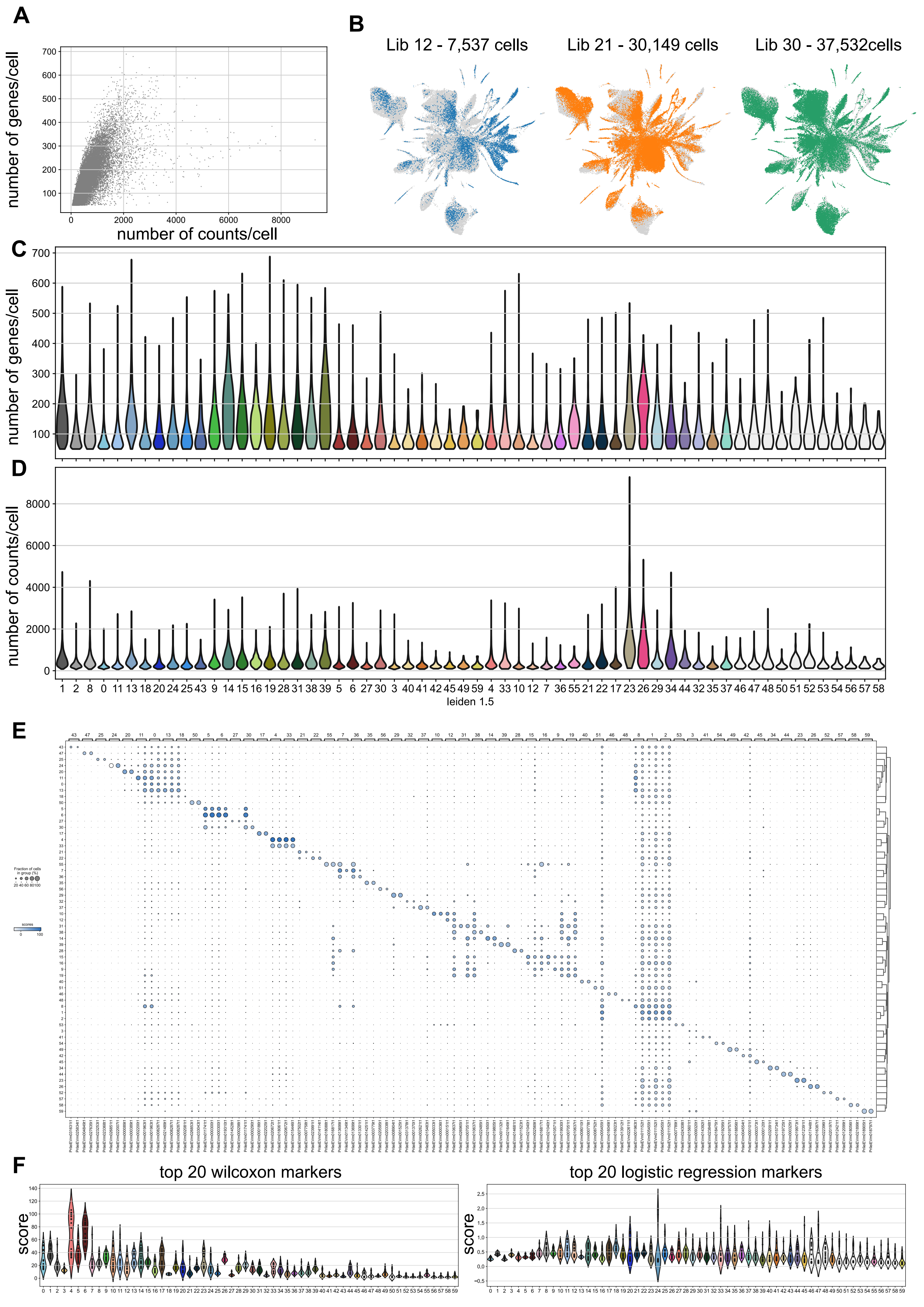
A
number of genes/cell
number of counts/cell
B
Lib 12 - 7,537 cells
Lib 21 - 30,149 cells
Lib 30 - 37,532cells
C
number of genes/cell
D
number of counts/cell
leiden 1.5
E
Fraction of cells in group (%)
scores
F
top 20 wilcoxon markers
top 20 logistic regression markers
score

Figure Supplementary 2: *Pristina leidyi* cell type atlas metrics.

A) Scatter plot of number of UMI counts per cell vs number of genes per cell.

B) UMAP visualisation of 75,218-cell *Pristina leidyi* single-cell transcriptomic cell atlas with cells coloured according to the experiment of origin, named Lib 12, Lib 21 and Lib 30.

C) Violin plots showing the distribution of genes detected per cell in each cluster.

D) Violin plots showing the distribution of UMI counts detected per cell in each cluster.

E) Dot Plot showing the expression of the top 2 markers detected by the Wilcoxon method in each cluster. Clusters are sorted according to hierarchical clustering. The values plotted in each dot are the fraction of cells in each cluster that express each marker (dot size) and the score of the marker (dot colour).

F) Score distribution of the top 20 markers of each cluster detected by the Wilcoxon method (left) and the logistic regression method (right).

Figure S3

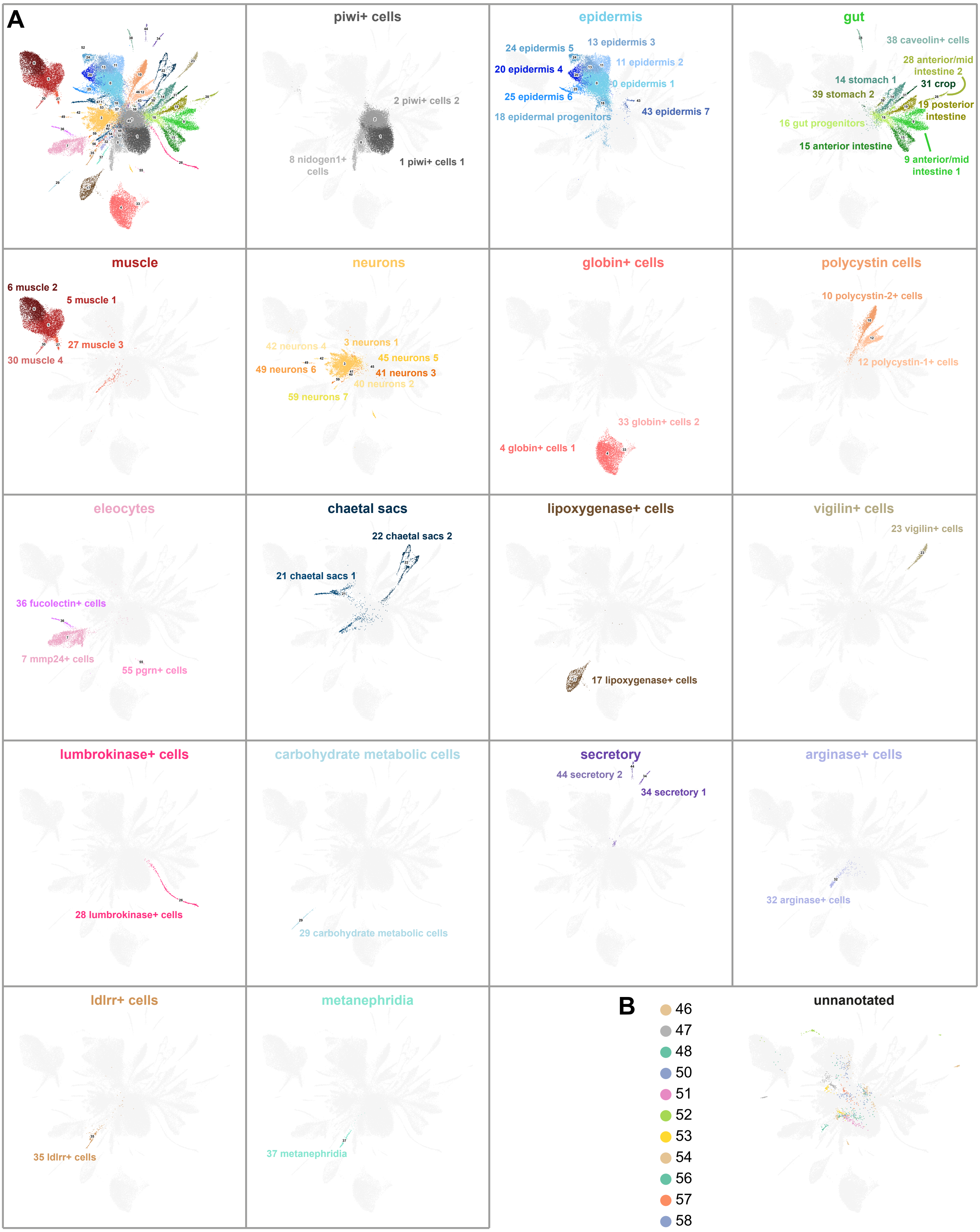
A
piwi+ cells
2 piwi+ cells 2
8 nidogen1+ cells
1 piwi+ cells 1
epidermis
24 epidermis 5
13 epidermis 3
11 epidermis 2
20 epidermis 4
0 epidermis 1
25 epidermis 6
43 epidermis 7
18 epidermal progenitors
gut
38 caveolin+ cells
28 anterior/mid intestine 2
14 stomach 1
31 crop
39 stomach 2
19 posterior intestine
16 gut progenitors
15 anterior intestine
9 anterior/mid intestine 1
muscle
6 muscle 2
5 muscle 1
27 muscle 3
30 muscle 4
neurons
42 neurons 4
3 neurons 1
45 neurons 5
49 neurons 6
41 neurons 3
40 neurons 2
59 neurons 7
globin+ cells
33 globin+ cells 2
4 globin+ cells 1
polycystin cells
10 polycystin-2+ cells
12 polycystin-1+ cells
eleocytes
36 fucolectin+ cells
7 mmp24+ cells
55 pgrn+ cells
chaetal sacs
22 chaetal sacs 2
21 chaetal sacs 1
lipoxygenase+ cells
17 lipoxygenase+ cells
vigilin+ cells
23 vigilin+ cells
lumbrokinase+ cells
28 lumbrokinase+ cells
carbohydrate metabolic cells
29 carbohydrate metabolic cells
secretory
44 secretory 2
34 secretory 1
arginase+ cells
32 arginase+ cells
ldlrr+ cells
35 ldlrr+ cells
metanephridia
37 metanephridia
B
46
47
48
50
51
52
53
54
56
57
58
unnanotated

Figure Supplementary 3: *Pristina leidyi* cell type atlas cell clusters and broad cell clusters.

A) UMAP visualisation of the 75,218-cell *Pristina leidyi* single-cell transcriptomic cell atlas with clusters coloured according to their cell type classification, subdivided by broad clusters.

B) UMAP visualisation of the 75,218-cell *Pristina leidyi* single-cell transcriptomic cell atlas with unannotated clusters highlighted.

Figure S4

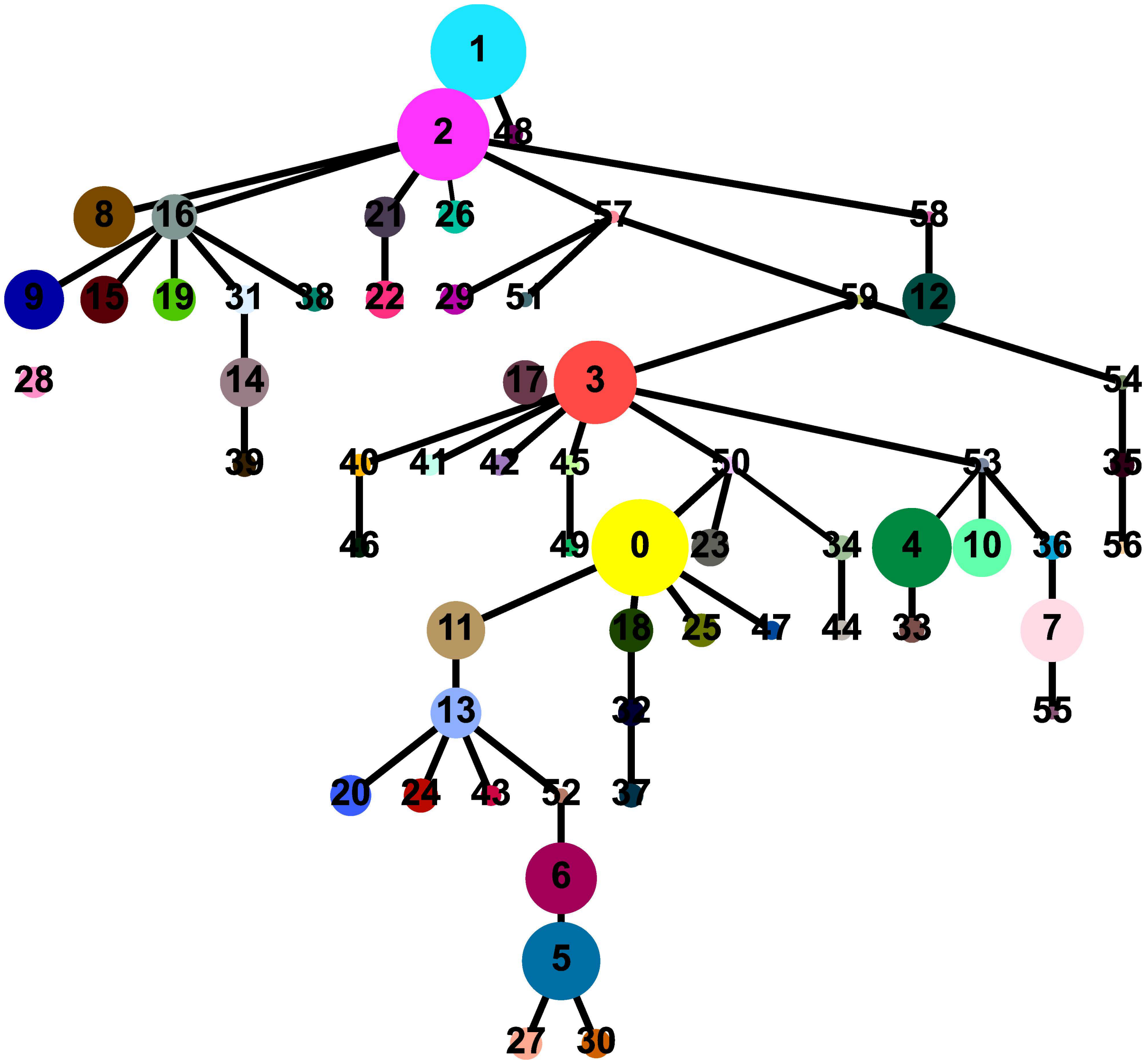

Figure Supplementary 4: Lineage reconstruction with unannotated clusters.

Lineage reconstruction abstracted graph showing the most probable path connecting the clusters. Each node corresponds to the cell clusters identified with the leiden algorithm. The size of nodes is proportional to the amount of cells in the cluster, and the thickness of the edges is proportional to the connectivity probabilities. This analysis includes the unannotated clusters (1,048 cells, 1.39% of the total dataset).

Figure S5

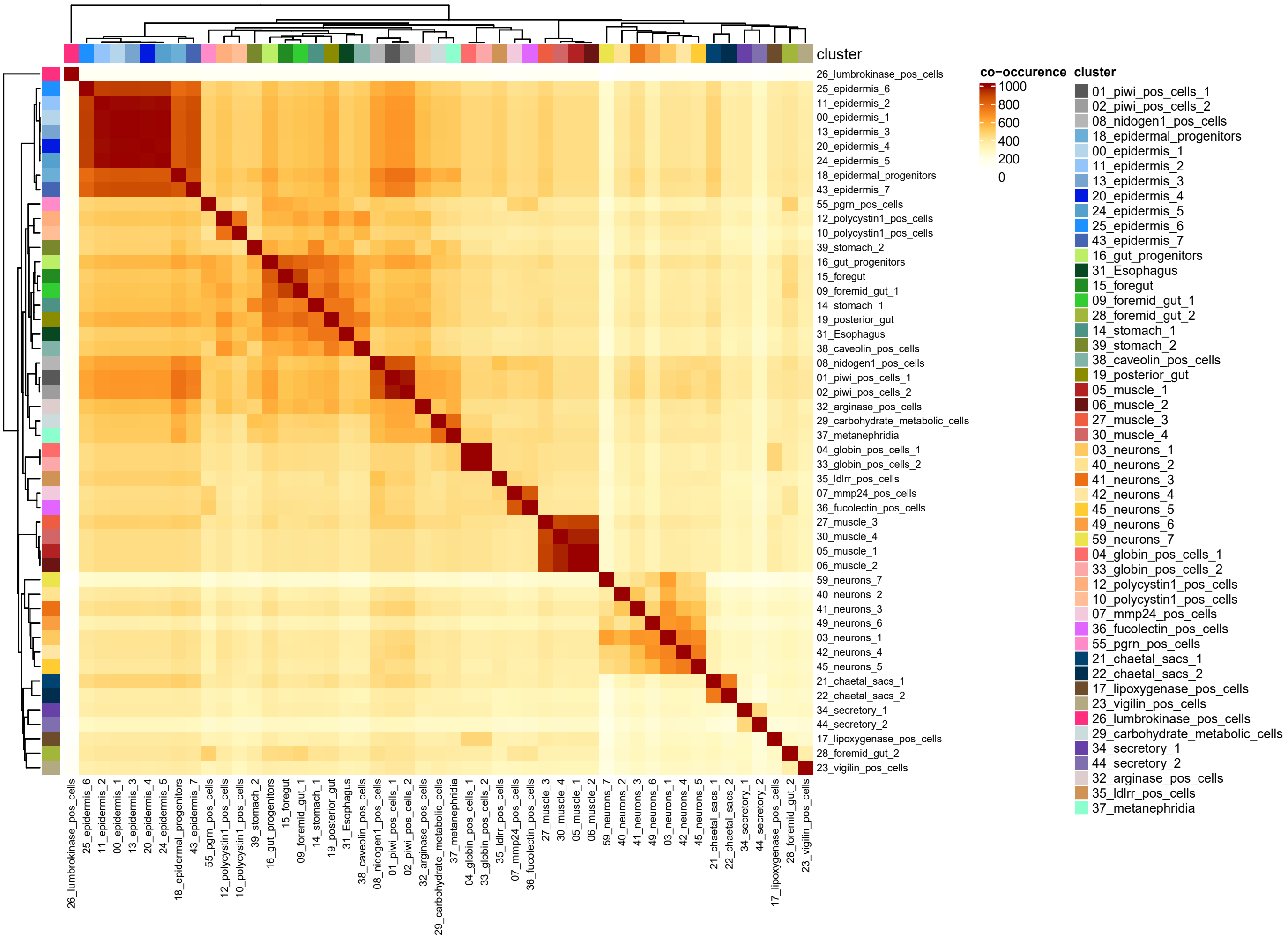

Figure Supplementary 5: Co-ocurrence analysis.

Cell cluster co-ocurrence matrix showing similarities between related cell types based on gene expression correlation.

Figure S6

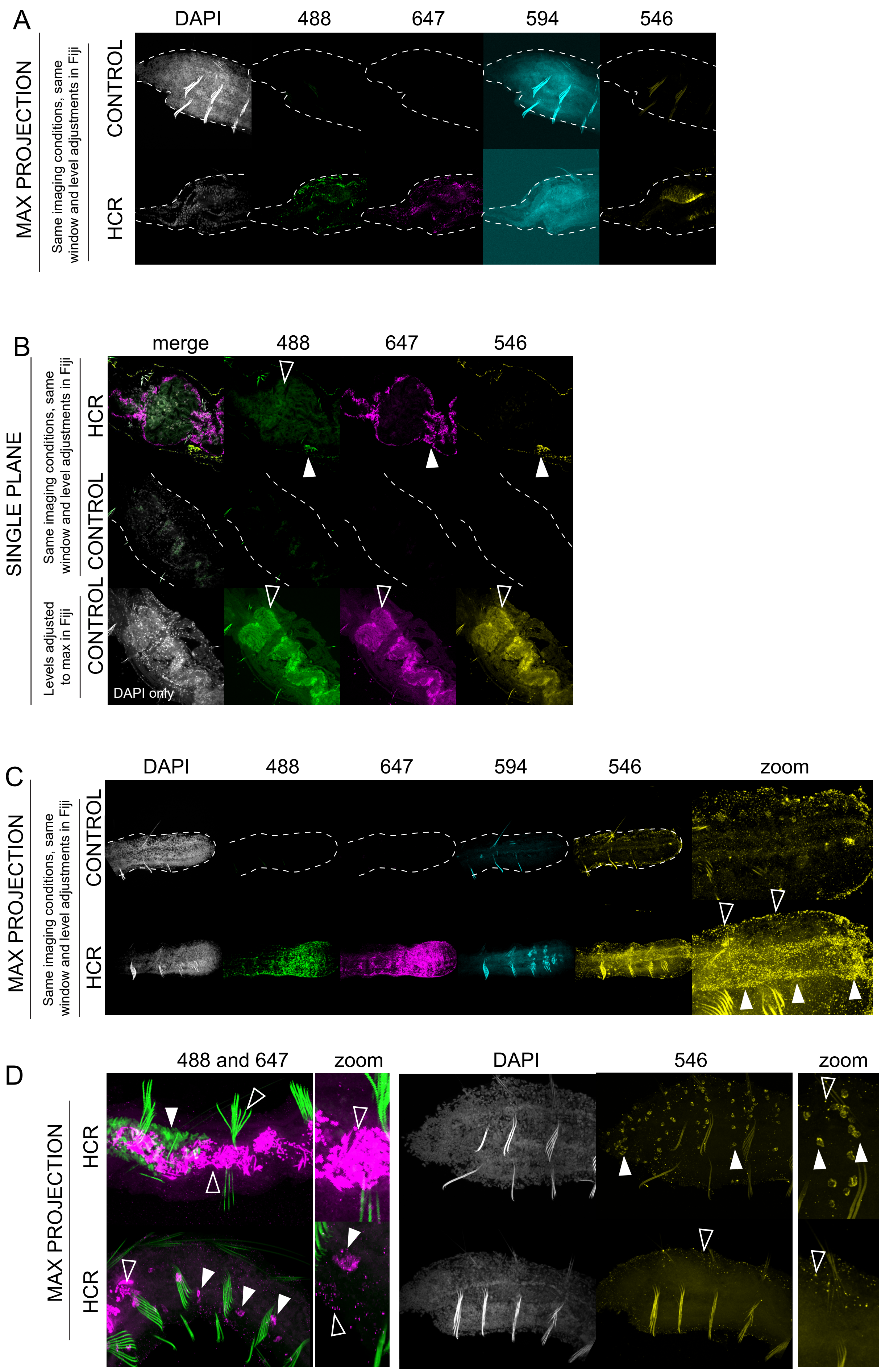
A
MAX PROJECTION
Same imaging conditions, same window and level adjustments in Fiji
CONTROL
HCR
DAPI
488
647
594
546
B
SINGLE PLANE
Same imaging conditions, same window and level adjustments in Fiji
Levels adjusted to max in Fiji
HCR
CONTROL
CONTROL
merge
488
647
546
DAPI only
C
MAX PROJECTION
Same imaging conditions, same window and level adjustments in Fiji
CONTROL
HCR
DAPI
488
647
594
546
zoom
D
MAX PROJECTION
HCR
HCR
488 and 647
zoom
DAPI
546
zoom

Figure Supplementary 6: Negative controls.

For all HCR experiments, controls that included only hairpin amplifiers but no probes were carried out, imaged using the same imaging conditions, and images were processed with the same settings as the HCR samples.

A) Panel shows maximum projections of Z-stacks in the head region of *Pristina* for a control and an HCR sample, when the same imaging and image processing conditions are applied. The head region typically did not have a lot of background or autofluorescence, except for chaetae, which typically show autofluorescence.

B) Single focal plane images of the gut region in HCR and control samples. Stomach region typically showed some background signal (hollow arrowheads) while the real signal was generally easy to distinguish (solid arrowheads) especially when signal-to-noise ratio was high. In the third row, background is shown when levels are adjusted to maximum in Fiji, but for the same control sample, if the image processing is done at the same levels as the HCR samples (second row), there is very little background.

C) Panel shows maximum projections of Z-stacks in the tail region for a control and an HCR sample, when the same imaging and image processing conditions are applied. When amplifiers with Alexa-546 fluorophore were used, we typically observed some background in the epidermal layer (hollow arrowheads), but the actual HCR signal was still possible to distinguish (solid arrowheads) (also see panel D).

D) Additional examples of autofluorescence and background signals. When the Alexa-647 fluorophore was used, gut content showed a high background (pink), therefore we avoided imaging the areas where there was high gut content. The chaetae (green, hollow arrowhead) is also shown as an example of intense autofluorescence. Nevertheless, the real signal was still possible to distinguish in these samples (solid arrowheads). On the right, we show another example of epidermal background (hollow arrowheads) versus actual signal (solid arrowheads) in the head region in different samples.

Figure S7

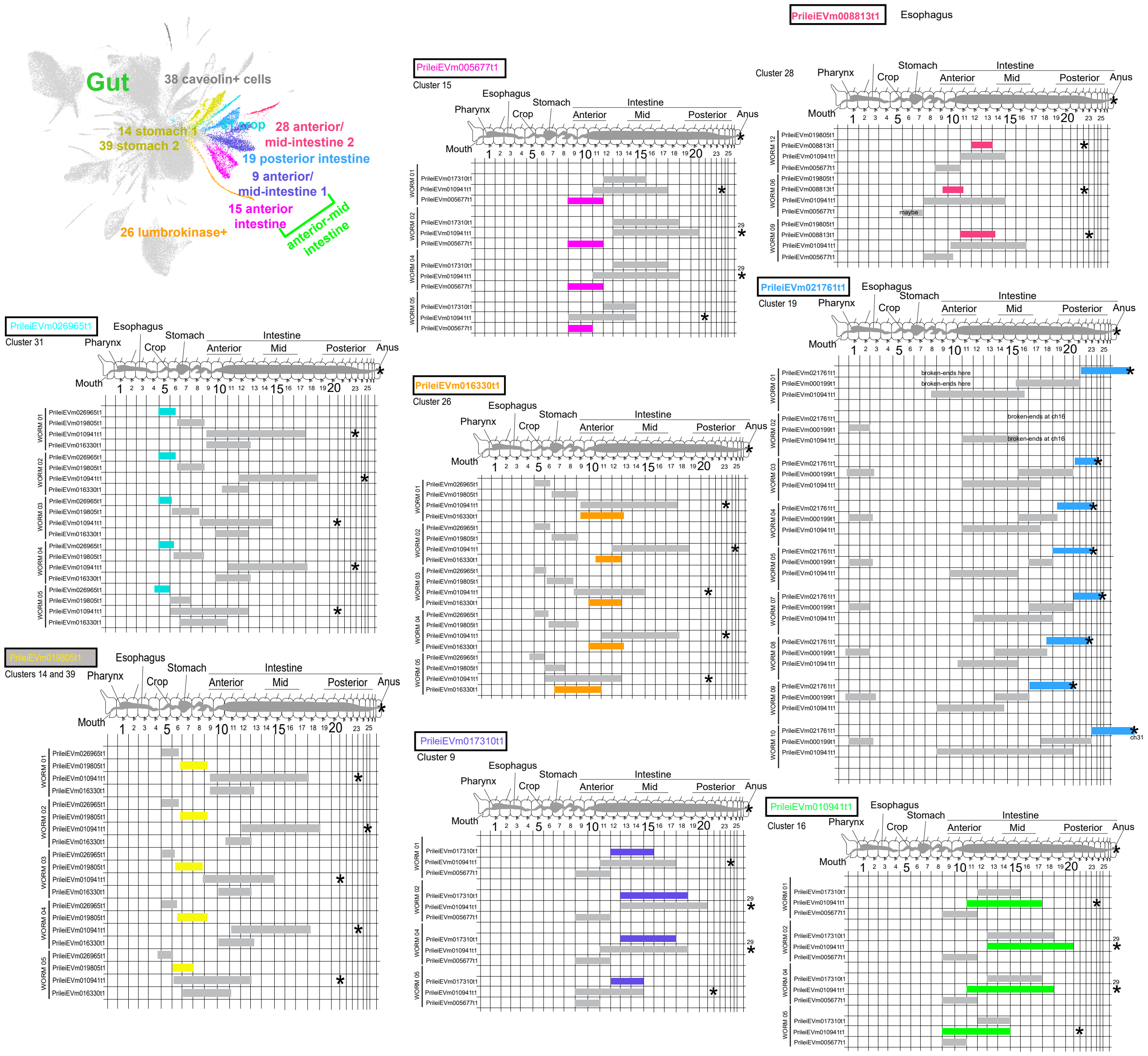

Figure Supplementary 7: Gut variations.

Detailed analyses showing all the samples analysed for the markers of gut clusters. Star denotes the tail end of the worm (anus). When there is a number next to the star, the number indicates the total number of segments for that particular worm (which was not possible to fit into the schematic because of length). 3 or 4 gut markers were tested in each set of worms, allowing analyses of multiple markers in a single sample. Each sample is indicated on the left (e.g. “Worm 01). Bars show the extent of expression observed for each marker.

Figure S8

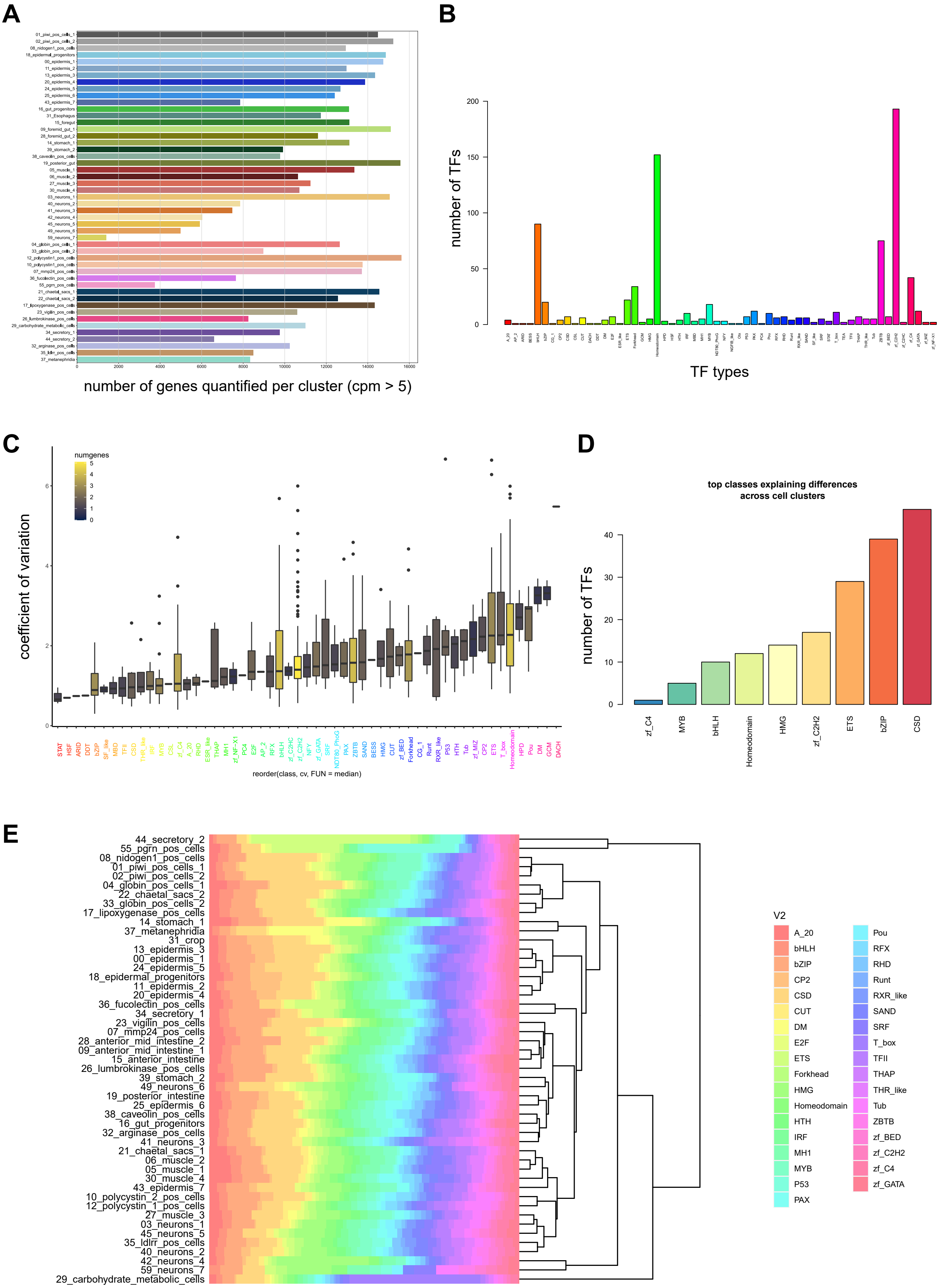

A
number of genes quantified per cluster (cpm > 5)
B
number of TFs
TF types
C
numgenes
coefficient of variation
reorder(class, cv, FUN = median)
STAT
HSF
ARID
DDT
bZIP
SF_like
MBD
TFII
CSD
THR_like
IRF
MYB
CSL
zf_C4
A_20
RHD
ESR_like
THAP
MH1
zf_NF-X1
PC4
E2F
AP_2
RFX
bHLH
zf_C2HC
zf_C2H2
NFY
zf_GATA
SRF
NDT80_PhoG
PAX
ZBTB
SAND
BESS
HMG
CUT
zf_BED
Forkhead
CG_1
Runt
RXR_like
P53
HTH
Tub
zf_MIZ
CP2
ETS
T_box
Homeodomain
HPD
Pou
DM
GCM
DACH
D
top classes explaining differences across cell clusters
number of TFs
zf_C4
MYB
bHLH
Homeodomain
HMG
zf_C2H2
ETS
bZIP
CSD
E
44_secretory_2
55_pgrn_pos_cells
08_nidogen1_pos_cells
01_piwi_pos_cells_1
02_piwi_pos_cells_2
04_globin_pos_cells_1
22_chaetal_sacs_2
33_globin_pos_cells_2
17_lipoxygenase_pos_cells
14_stomach_1
37_metanephridia
31_crop
13_epidermis_3
00_epidermis_1
24_epidermis_5
18_epidermal_progenitors
11_epidermis_2
20_epidermis_4
36_fucolectin_pos_cells
34_secretory_1
23_vigilin_pos_cells
07_mmp24_pos_cells
28_anterior_mid_intestine_2
09_anterior_mid_intestine_1
15_anterior_intestine
26_lumbrokinase_pos_cells
39_stomach_2
49_neurons_6
19_posterior_intestine
25_epidermis_6
38_caveolin_pos_cells
16_gut_progenitors
32_arginase_pos_cells
41_neurons_3
21_chaetal_sacs_1
06_muscle_2
05_muscle_1
30_muscle_4
43_epidermis_7
10_polycystin_2_pos_cells
12_polycystin_1_pos_cells
27_muscle_3
03_neurons_1
45_neurons_5
35_ldlrr_pos_cells
40_neurons_2
42_neurons_4
59_neurons_7
29_carbohydrate_metabolic_cells
V2
A_20
bHLH
bZIP
CP2
CSD
CUT
DM
E2F
ETS
Forkhead
HMG
Homeodomain
HTH
IRF
MH1
MYB
P53
PAX
Pou
RFX
RHD
Runt
RXR_like
SAND
SRF
T_box
TFII
THAP
THR_like
Tub
ZBTB
zf_BED
zf_C2H2
zf_C4
zf_GATA

Figure Supplementary 8: Pseudobulk Transcription Factor Analysis.

A) Barplot showing the number of genes quantified with more than 5 UMI counts per million (cpm) per cluster in a pseudobulk analysis, showing that most clusters have > 4000 genes quantified, with a mean of 11,117 genes per cluster.

B) Barplot showing the number of TFs per TF type.

C) Boxplot showing the coefficient of variation distribution for each TF type. Boxes represent lower quartile, median, and upper quartile; whiskers represent 1.5 x interquartile ranges; dots represent outliers.

D) Barplot showing the top TF classes based on the number of instances where gene expression of a given TF class was found to be significant in explaining differences between cell clusters (ANOVA, Tukey test comparison of means).

E) (Left) Barplot showing predominance of different TF classes in the transcriptomic profile of different cell clusters, measured as number of CPMs per gene and per class. Each colour represents a transcription factor class. (Right) clustering of cell clusters based on similarities in the transcriptomic profile of TF classes.

Figure S9

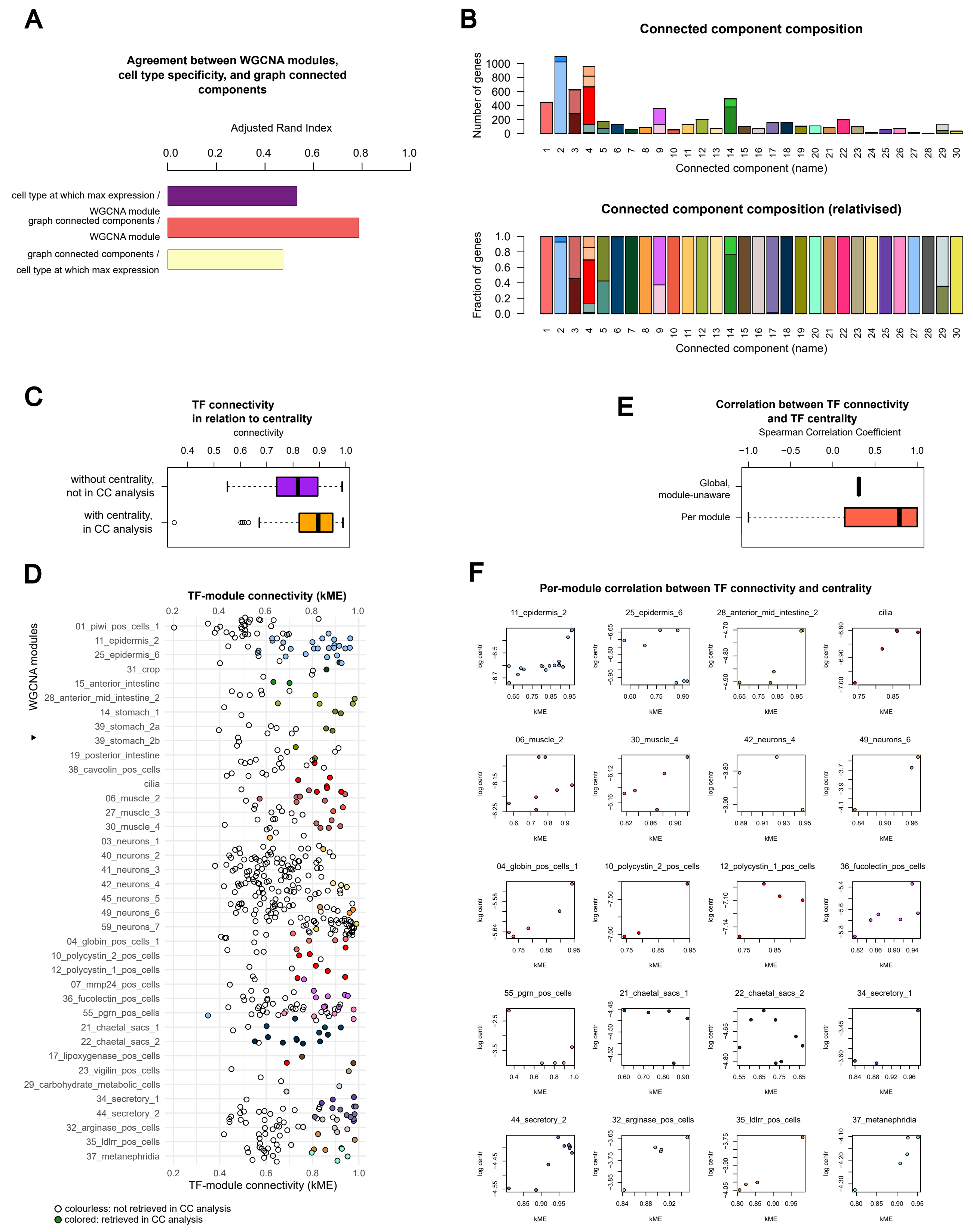

Figure Supplementary 9: WGCNA graph analysis.

A) Barplot showing the adjusted Rand index metric to assess the agreement between several gene classifications: genes assigned to WGCNA modules vs genes assigned to cell types based on

maximum expression, genes assigned to WGCNA modules vs genes assigned to graph connected components, and genes assigned to graph connected components vs genes assigned to cell types based on maximum expression.

B) (up) barplot showing the WGCNA module composition of each connected component. Colour indicates WGCNA module as shown in Fig. 4F,G. (down) similar as upper barplot but relativised to show relative frequencies.

C) Boxplot showing the connectivity (kME) values of TF genes that did or did not survive the CC analysis and thus have associated centrality values. Boxes represent lower quartile, median, and upper quartile; whiskers represent 1.5 x interquartile ranges; dots represent outliers.

D) Strip chart showing kME of TFs in the WGCNA module analysis, highlighting those with associated centrality.

E) Boxplot showing the differences in correlation when taking all kME and centrality values without regard to module membership (above value) vs when calculating separate correlations between kME and centrality for each module separately. Boxes represent lower quartile, median, and upper quartile; whiskers represent 1.5 x interquartile ranges; dots represent outliers.

F) Scatter plots of the kME and the centrality values of each TF in each of their respective modules.

Figure S10

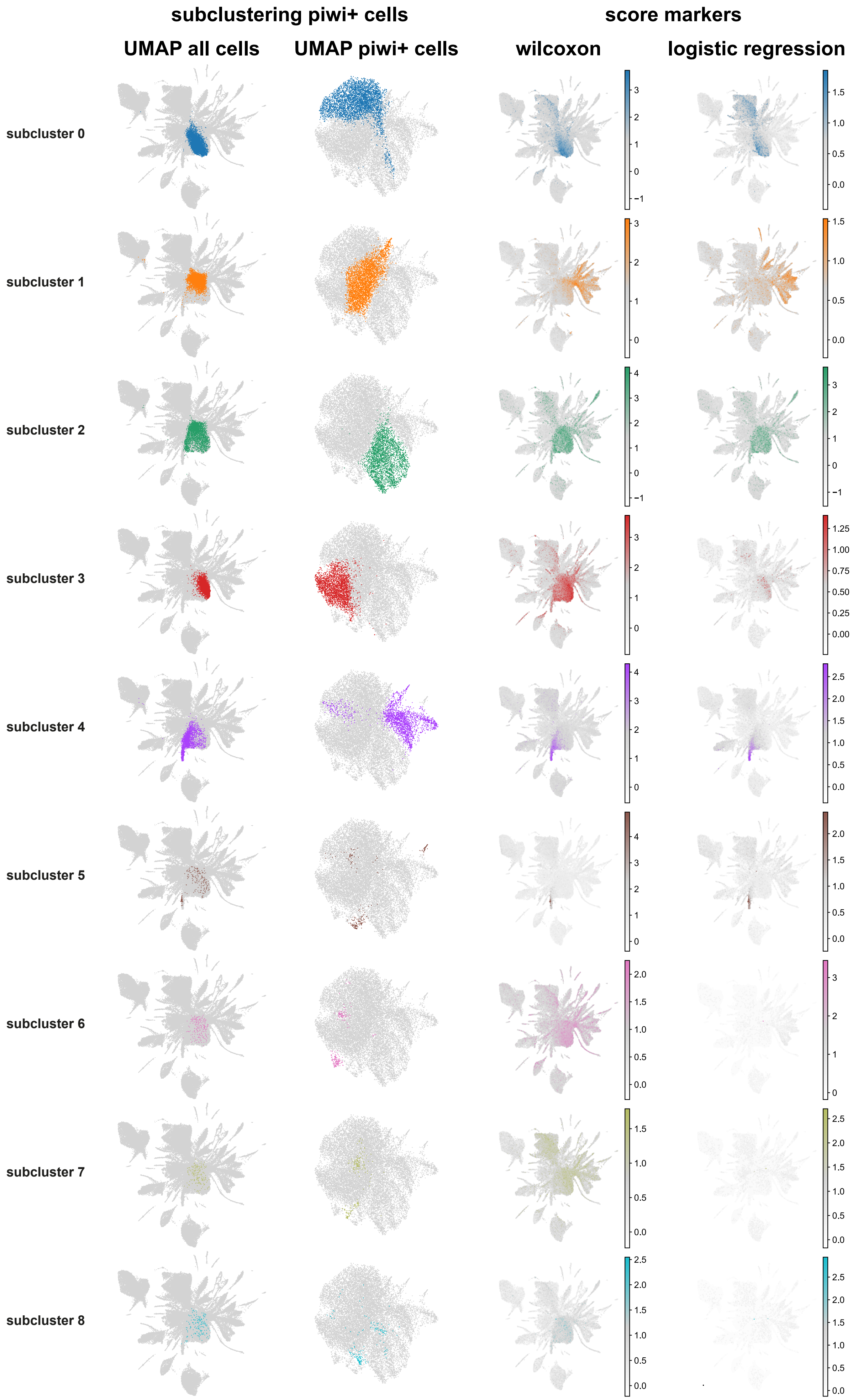
subclustering piwi+ cells
score markers
UMAP all cells
UMAP piwi+ cells
wilcoxon
logistic regression
subcluster 0
subcluster 1
subcluster 2
subcluster 3
subcluster 4
subcluster 5
subcluster 6
subcluster 7
subcluster 8

Figure Supplementary 10: Subclustering of *piwi*+ cells.

UMAP visualisation of *piwi*+ subclusters in the 75,218 and the 16,247 datasets containing all cells and only *piwi*+ cell clusters, respectively, and score UMAP plots of markers of *piwi*+ subclusters. Markers were calculated using the Wilcoxon and the Logistic Regression methods.

Supplementary References

1 Nyberg, K. G., Conte, M. A., Kostyun, J. L., Forde, A. & Bely, A. E. Transcriptome characterization via 454 pyrosequencing of the annelid Pristina leidyi, an emerging model for studying the evolution of regeneration. *BMC Genomics* **13**, 287, (2012).

2 Gilbert, D. G. Longest protein, longest transcript or most expression, for accurate gene reconstruction of transcriptomes? *bioRxiv*, 829184, (2019).

3 Cantalapiedra, C. P., Hernandez-Plaza, A., Letunic, I., Bork, P. & Huerta-Cepas, J. eggNOG-mapper v2: Functional Annotation, Orthology Assignments, and Domain Prediction at the Metagenomic Scale. *Mol Biol Evol* **38**, 5825-5829, (2021).

4 Buchfink, B., Reuter, K. & Drost, H. G. Sensitive protein alignments at tree-of-life scale using DIAMOND. *Nat Methods* **18**, 366-368, (2021).

5 Garcia-Castro, H. *et al.* ACME dissociation: a versatile cell fixation-dissociation method for single-cell transcriptomics. *Genome Biol* **22**, 89, (2021).

6 Rosenberg, A. B. *et al.* Single-cell profiling of the developing mouse brain and spinal cord with split-pool barcoding. *Science* **360**, 176-182, (2018).

7 Wolock, S. L., Lopez, R. & Klein, A. M. Scrublet: Computational Identification of Cell Doublets in Single-Cell Transcriptomic Data. *Cell Syst* **8**, 281-291 e289, (2019).

8 Bernstein, N. J. *et al.* Solo: Doublet Identification in Single-Cell RNA-Seq via Semi-Supervised Deep Learning. *Cell Syst* **11**, 95-101 e105, (2020).

9 Wolf, F. A. *et al.* PAGA: graph abstraction reconciles clustering with trajectory inference through a topology preserving map of single cells. *Genome Biol* **20**, 59, (2019).

10 Levy, S. *et al.* A stony coral cell atlas illuminates the molecular and cellular basis of coral symbiosis, calcification, and immunity. *Cell* **184**, 2973-2987 e2918, (2021).

11 Langfelder, P. & Horvath, S. WGCNA: an R package for weighted correlation network analysis. *BMC Bioinformatics* **9**, 559, (2008).

12 Zhang, B. & Horvath, S. A general framework for weighted gene co-expression network analysis. *Stat Appl Genet Mol Biol* **4**, Article17, (2005).

13 Aibar, S. *et al.* SCENIC: single-cell regulatory network inference and clustering. *Nat Methods* **14**, 1083-1086, (2017).

14 Badia, I. M. P. *et al.* Gene regulatory network inference in the era of single-cell multi-omics. *Nat Rev Genet* **24**, 739-754, (2023).

15 Mercatelli, D., Scalambra, L., Triboli, L., Ray, F. & Giorgi, F. M. Gene regulatory network inference resources: A practical overview. *Biochim Biophys Acta Gene Regul Mech* **1863**, 194430, (2020).

16 Xu, Q. *et al.* ANANSE: an enhancer network-based computational approach for predicting key transcription factors in cell fate determination. *Nucleic Acids Res* **49**, 7966-7985, (2021).
