# Supplementary material for: Annelid adult cell type diversity and their pluripotent cellular origins
Source: Nat Commun. 2024 Apr 12;15:3194. doi: 10.1038/s41467-024-47401-6 (PMC11014941; doi:10.1038/s41467-024-47401-6)
Supplement: Supplementary file 3 — Description of Additional Supplementary Files [file 41467_2024_47401_MOESM3_ESM.pdf]

Description of Additional Supplementary Files

File Name: Supplementary Data 1
Description: **EggNOG and Diamond Blast Annotation.**

File Name: Supplementary Data 2
Description: **Preprocessing parameter space exploration.**
UMAP visualisation of the 80,387 cells preprocessed with different parameters for minimum gene UMI counts (30-100), maximum number of genes (300-1000), maximum number of UMI counts (500-1200), number of top highly variable genes (4,000-22,000), number of neighbors (15-85), and number of principal components (15-145). For each parameter, UMAP feature plots of diagnostic markers PrileiEVm023936t1 (piwi+ cells), PrileiEVm008309t1 (epidermis), PrileiEVm011741t1 (muscle), PrileiEVm021316t1 (gut), PrileiEVm022250t1 (gut), PrileiEVm000325t1 (neurons), PrileiEVm013699t1 (eleocytes), and PrileiEVm020595t1 (unnanotated).

File Name: Supplementary Data 3
Description: **Cluster Wilcoxon markers and Diamond Blast annotations.**
Marker genes per cluster calculated using the Wilcoxon method with maximum p-values of 0.05.

File Name: Supplementary Data 4
Description: **Cluster Logistic Regression markers and Diamond Blast annotations.**
Top 30 markers per cluster calculated using the Logistic Regression method.

File Name: Supplementary Data 5
Description: **Pristina leidyi cell cluster markers.**
UMAP visualisation of 75,218-cell Pristina leidyi single-cell transcriptomic cell atlas. In the top left panel of each page each cluster is coloured. In the remaining plots, UMAP feature plots of markers of the top 8 common markers among the Wilcoxon and the Logistic Regression methods. If there are less than 8 common markers the plots only display the UMAP visualisation without any feature gene expression.

File Name: Supplementary Data 6
Description: **HCR in situ probes.**

File Name: Supplementary Data 7
Description: **WGCNA modules.**

File Name: Supplementary Data 8
Description: **WGCNA modules GO terms.**

File Name: Supplementary Data 9
Description: **Transcription Factor annotations.**

File Name: Supplementary Data 10
Description: **piwi+ cells COGs.**

File Name: Supplementary Data 11
Description: **Top 200 TFs expressed in piwi+ cells.**
Expression (in log-ratio of counts in a given broad cell type vs piwi+ cells) of the top 200 variable (CV > 1) transcription factors in Pristina leidyi; as well as the top hit in the DIAMOND annotation. See Methods for further details.

File Name: Supplementary Data 12
Description: **Top 200 TFs expressed in piwi+ cells UMAP visualisation.**

File Name: Supplementary Data 13
Description: **Transcriptomic profile of piwi+ cells and Diamond Blast annotations.**

File Name: Supplementary Data 14
Description: **Annotation of Pristina epigenetic factor homologues.**
