# Supplementary material for: Annelid adult cell type diversity and their pluripotent cellular origins
Source: Nat Commun. 2024 Apr 12;15:3194. doi: 10.1038/s41467-024-47401-6 (PMC11014941; doi:10.1038/s41467-024-47401-6)

minimum gene counts

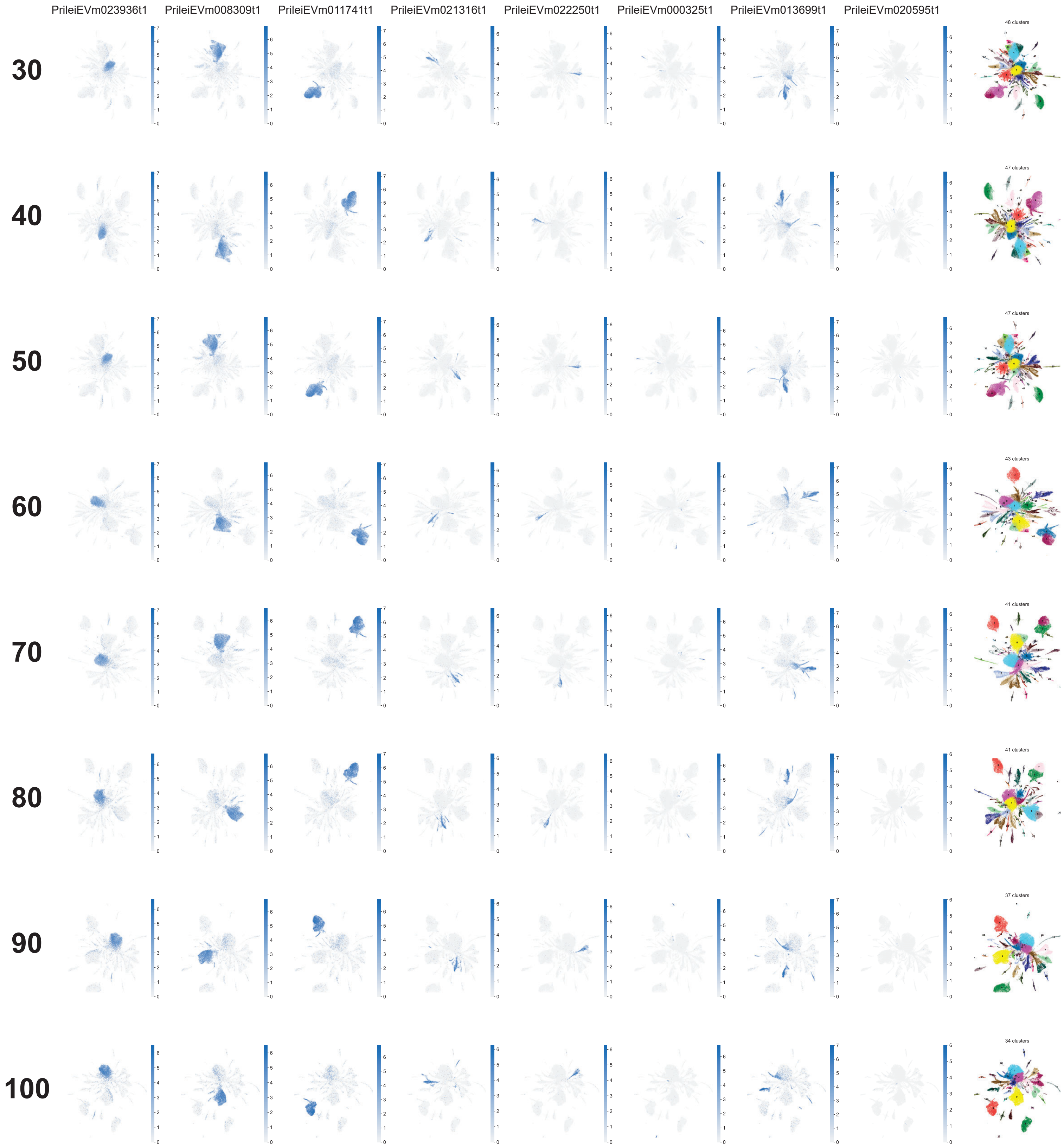

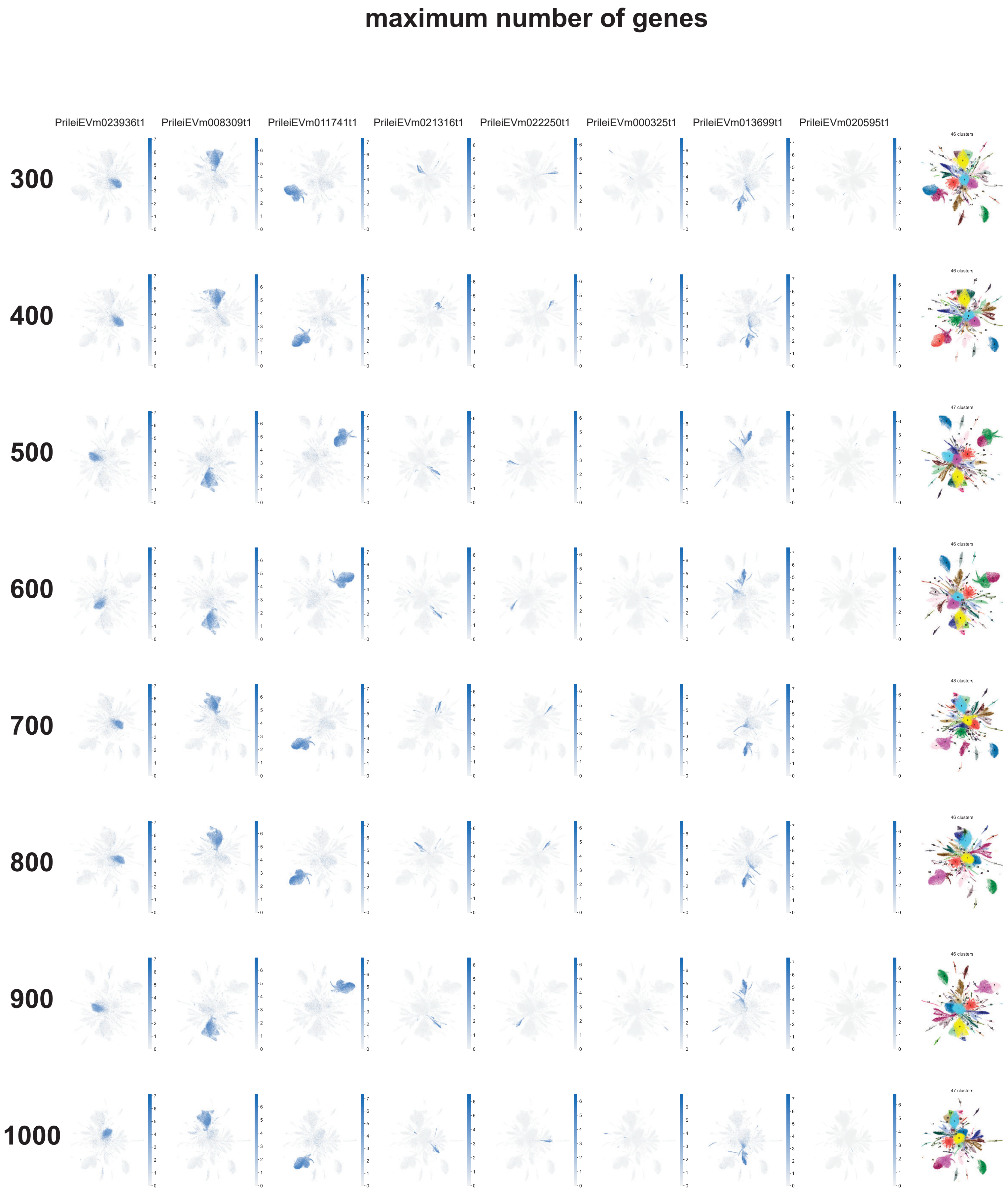
maximum number of genes
PrileiEVm023936t1
PrileiEVm008309t1
PrileiEVm011741t1
PrileiEVm021316t1
PrileiEVm022250t1
PrileiEVm000325t1
PrileiEVm013699t1
PrileiEVm020595t1
300
400
500
600
700
800
900
1000
46 clusters
47 clusters
48 clusters

maximum number of counts

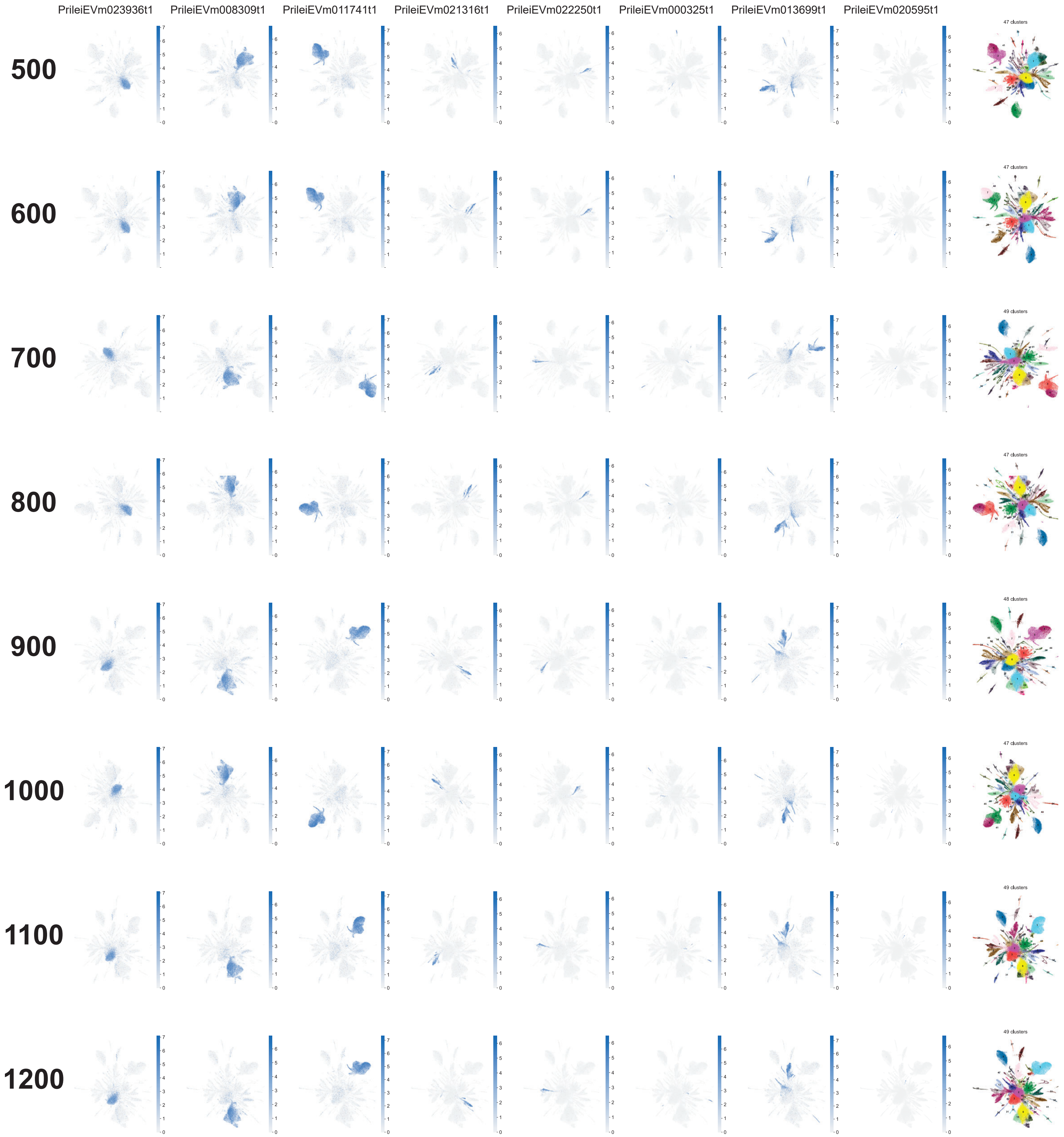

number of top highly variable genes

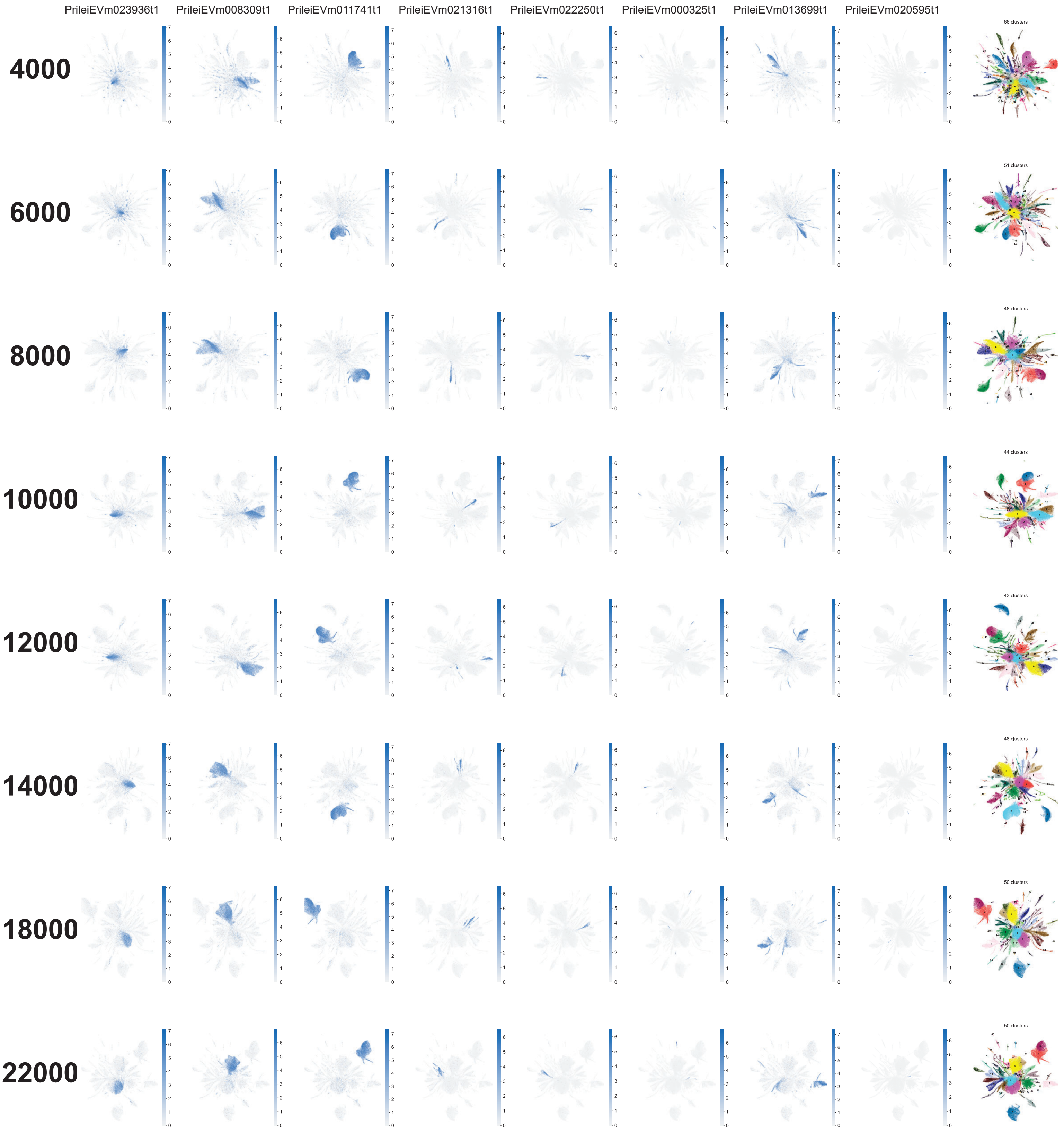

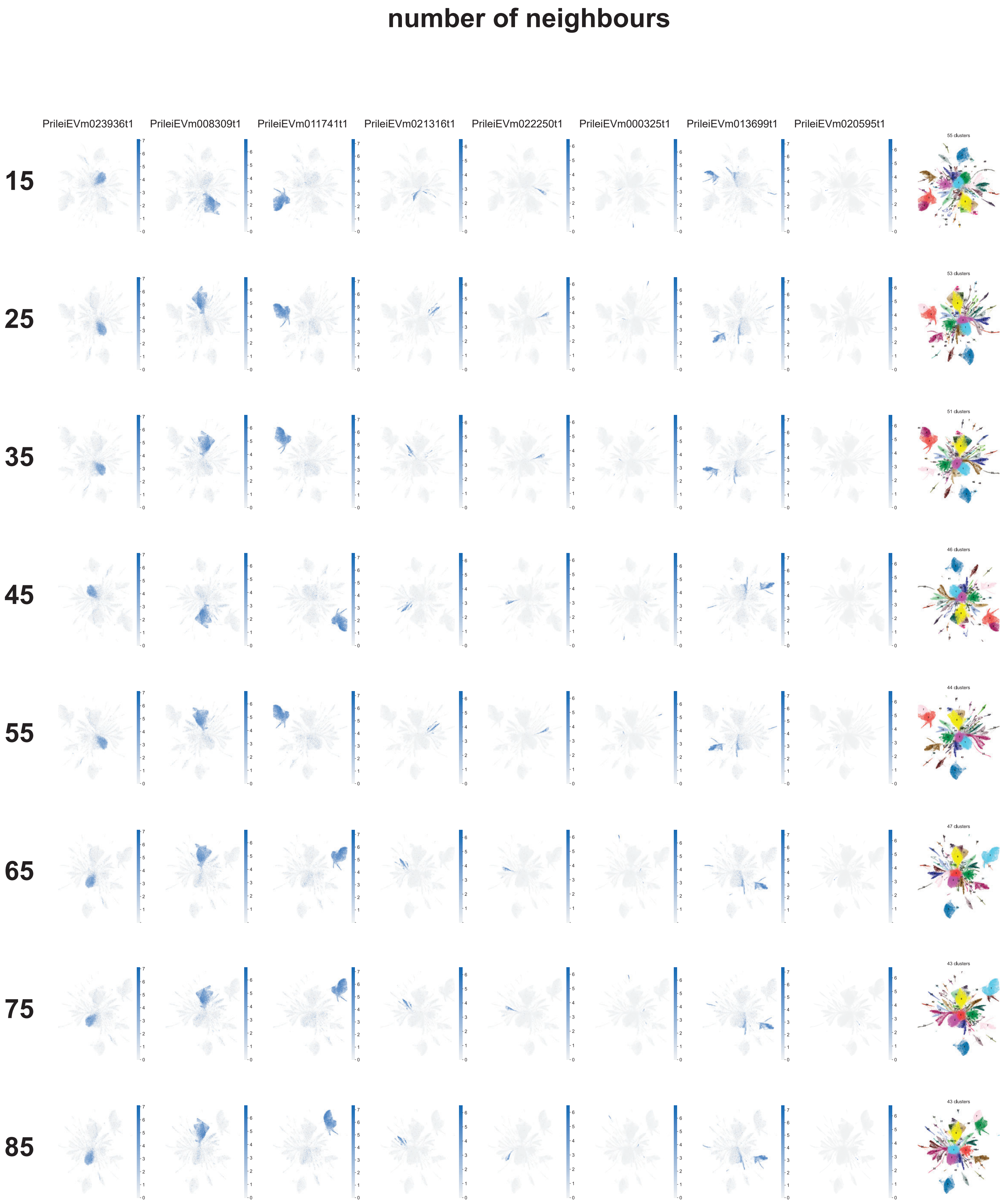
number of neighbours
PrileiEVm023936t1
PrileiEVm008309t1
PrileiEVm011741t1
PrileiEVm021316t1
PrileiEVm022250t1
PrileiEVm000325t1
PrileiEVm013699t1
PrileiEVm020595t1
15
55 clusters
25
53 clusters
35
51 clusters
45
46 clusters
55
44 clusters
65
47 clusters
75
43 clusters
85
43 clusters

number of principal components

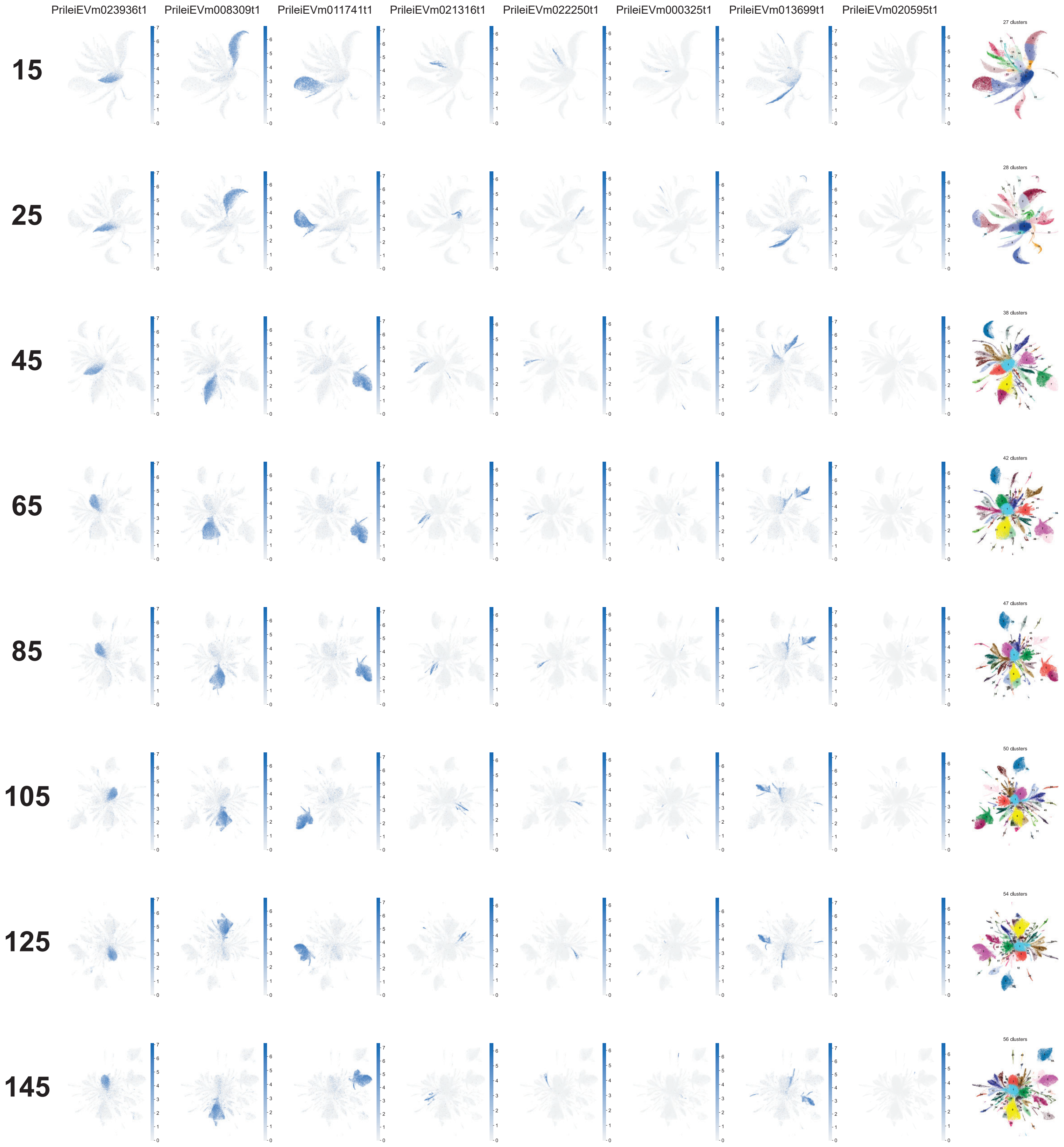

Supplement: Supplementary file 5 — Supplementary Data 2 [file 41467_2024_47401_MOESM5_ESM.pdf]
