# Supplementary figures and images for: Annelid adult cell type diversity and their pluripotent cellular origins
Source: Nat Commun. 2024 Apr 12;15:3194. doi: 10.1038/s41467-024-47401-6 (PMC11014941; doi:10.1038/s41467-024-47401-6)

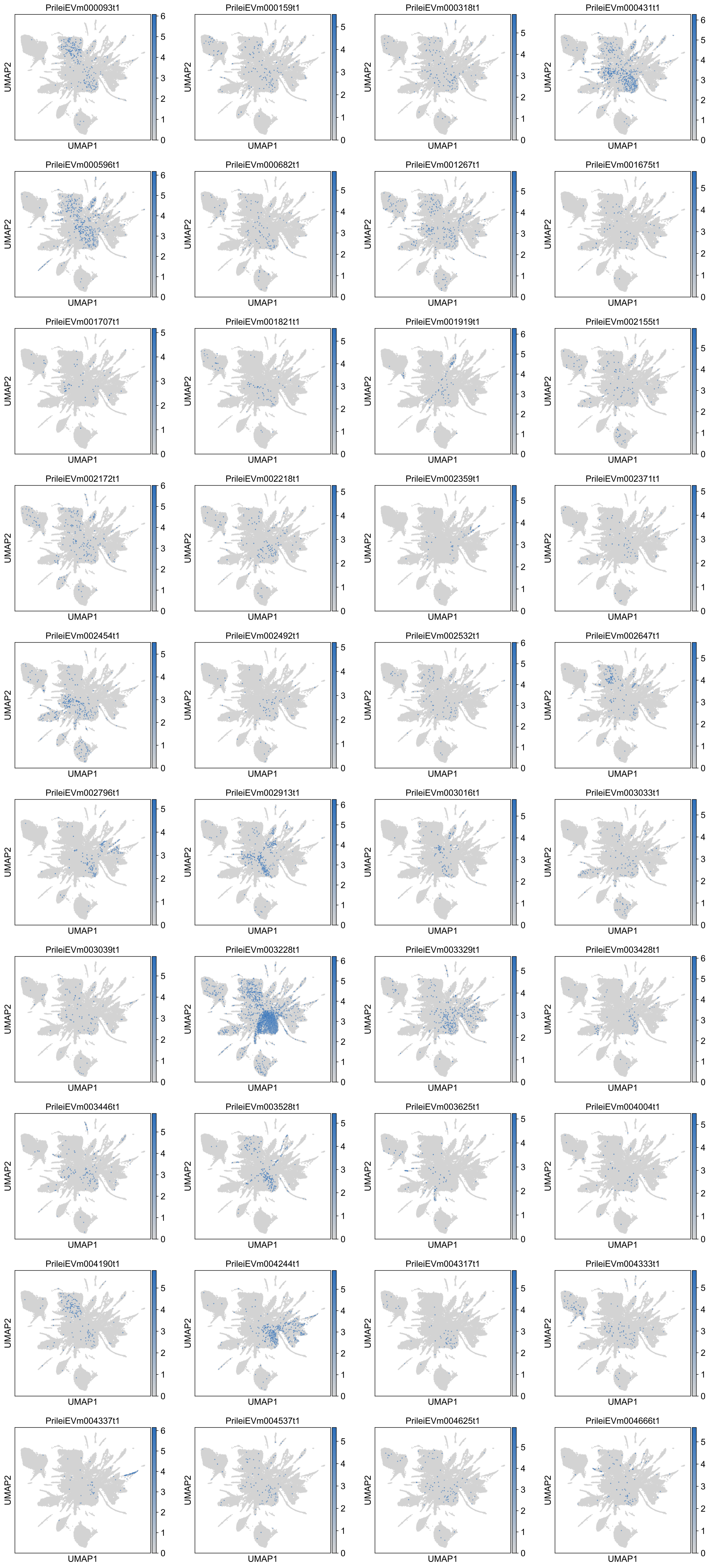

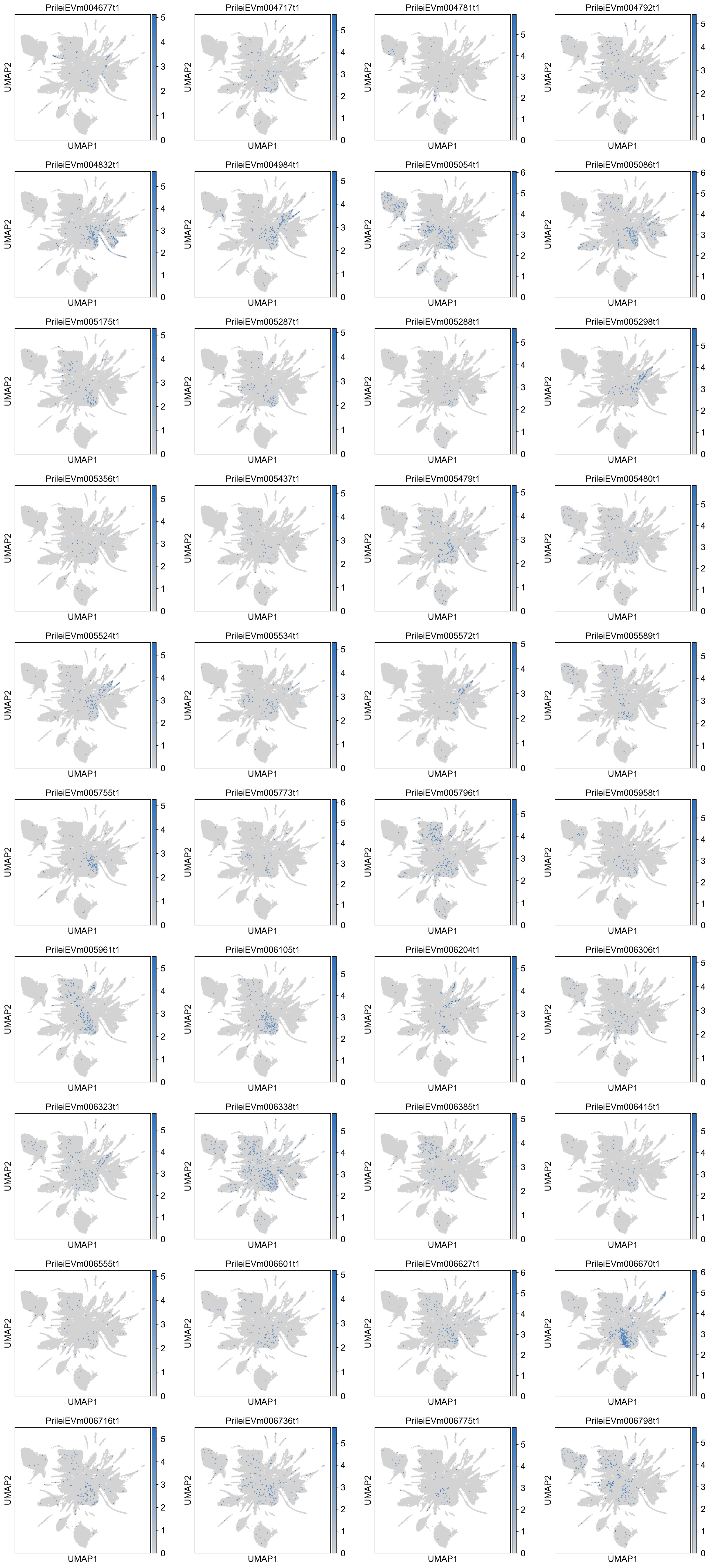

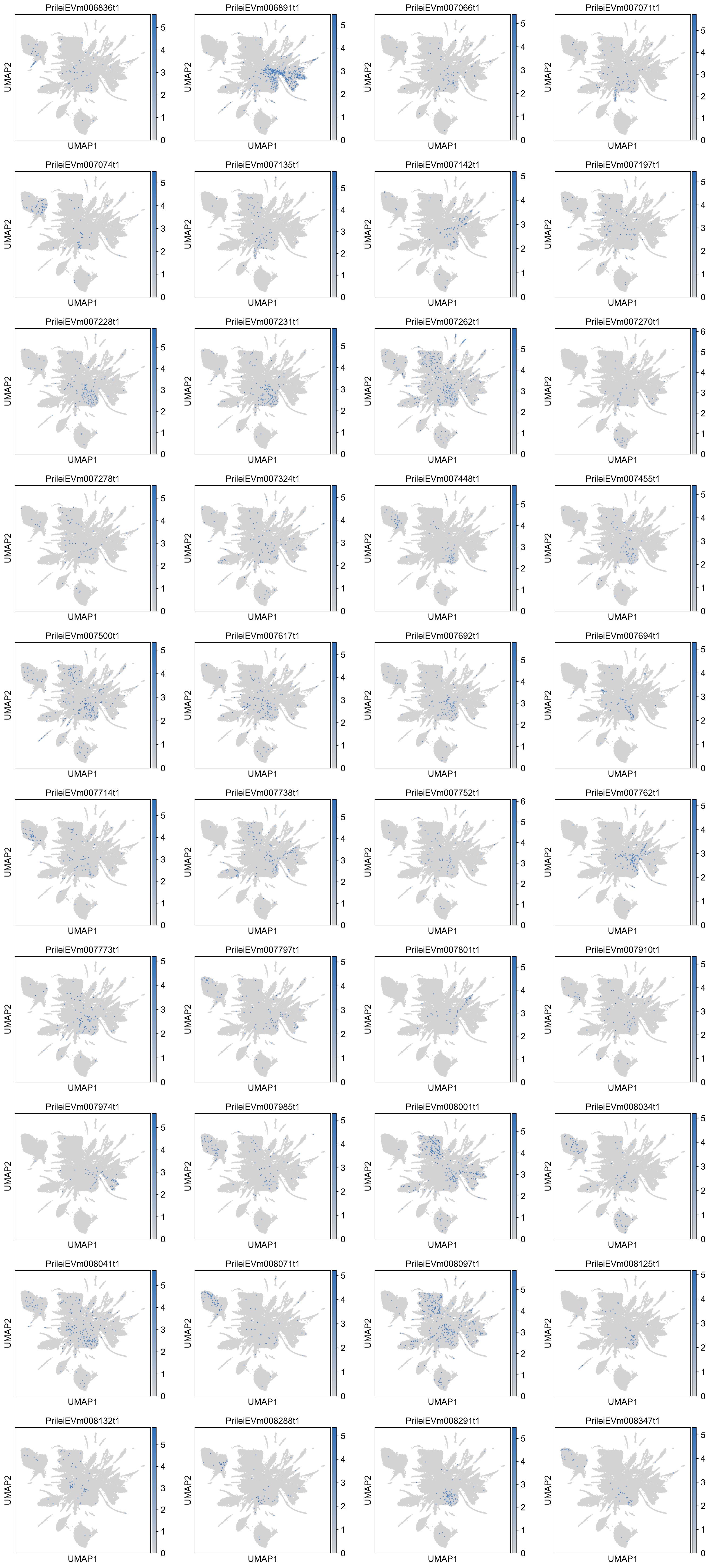

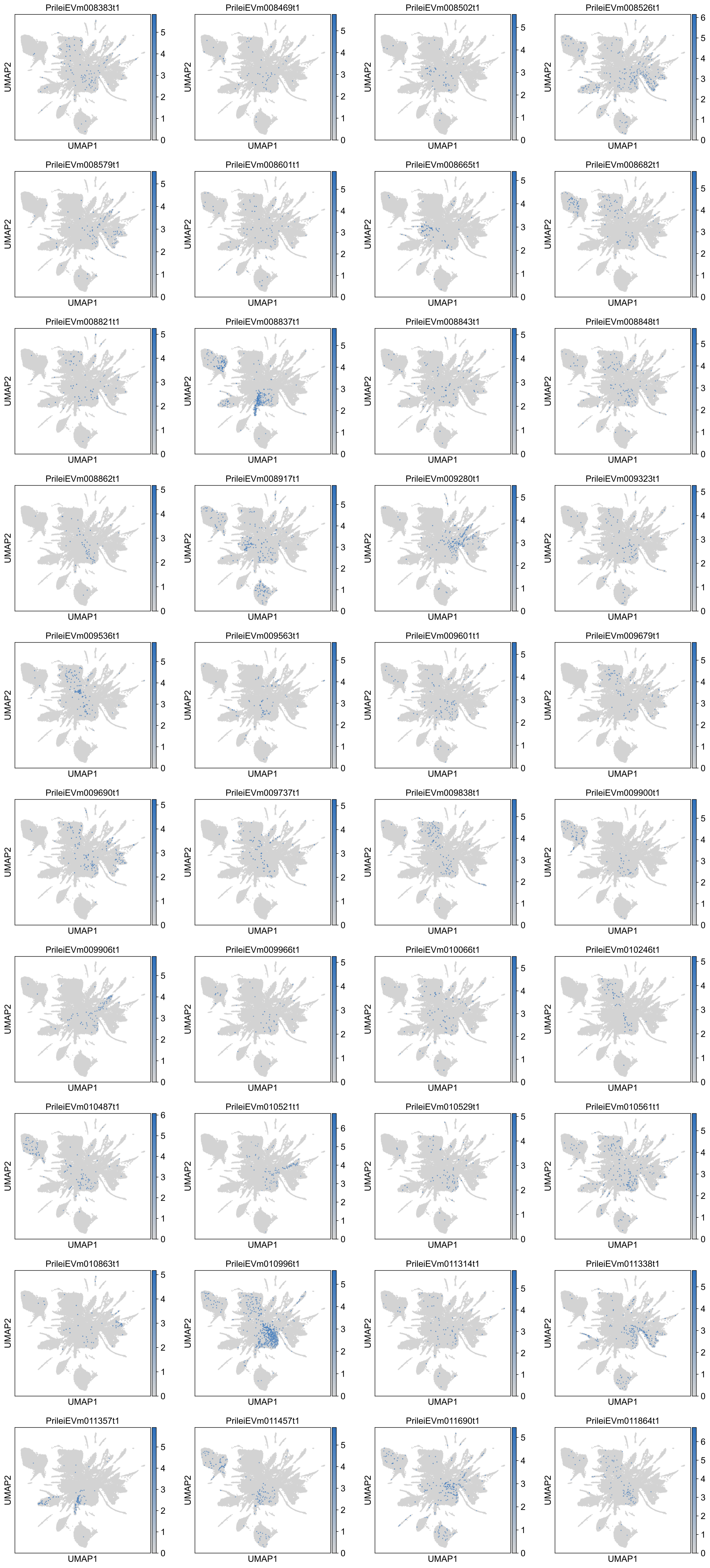

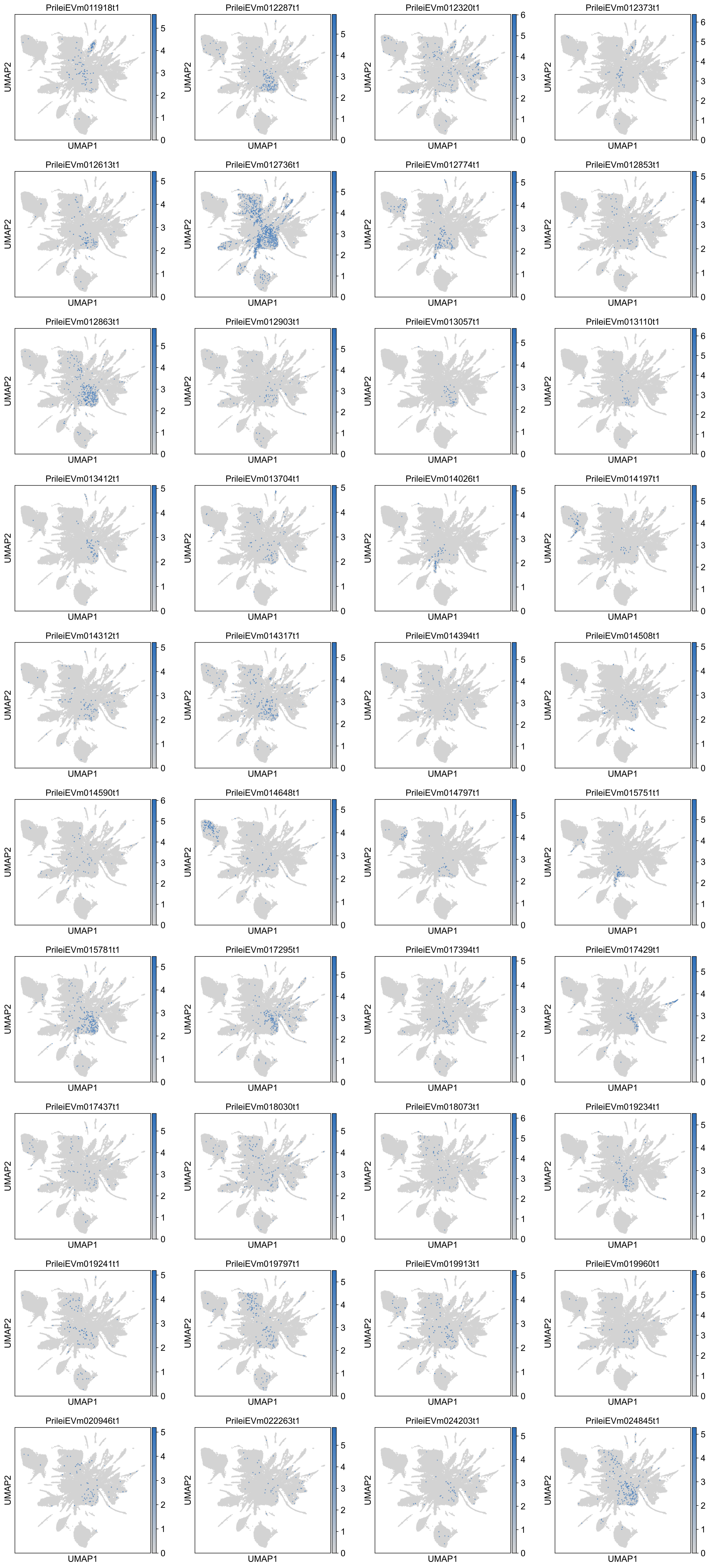

Supplement: Supplementary file 15 — Supplementary Data 12 [file 41467_2024_47401_MOESM15_ESM.pdf]
