# Supplementary material for: Annelid adult cell type diversity and their pluripotent cellular origins
Source: Nat Commun. 2024 Apr 12;15:3194. doi: 10.1038/s41467-024-47401-6 (PMC11014941; doi:10.1038/s41467-024-47401-6)
Supplement: Supplementary file 18 — Reporting Summary [file 41467_2024_47401_MOESM18_ESM.pdf]

nature portfolio

Corresponding author(s): Jordi Solana, Patricia Álvarez-Campos, B. Duygu Özpolat

Last updated by author(s): 14/02/2024

Reporting Summary

Nature Portfolio wishes to improve the reproducibility of the work that we publish. This form provides structure for consistency and transparency in reporting. For further information on Nature Portfolio policies, see our Editorial Policies and the Editorial Policy Checklist.

Statistics

For all statistical analyses, confirm that the following items are present in the figure legend, table legend, main text, or Methods section.

n/a	Confirmed	
☐	☒	The exact sample size (*n*) for each experimental group/condition, given as a discrete number and unit of measurement
☐	☒	A statement on whether measurements were taken from distinct samples or whether the same sample was measured repeatedly
☐	☒	The statistical test(s) used AND whether they are one- or two-sided<br>*Only common tests should be described solely by name; describe more complex techniques in the Methods section.*
☒	☐	A description of all covariates tested
☐	☒	A description of any assumptions or corrections, such as tests of normality and adjustment for multiple comparisons
☐	☒	A full description of the statistical parameters including central tendency (e.g. means) or other basic estimates (e.g. regression coefficient) AND variation (e.g. standard deviation) or associated estimates of uncertainty (e.g. confidence intervals)
☐	☒	For null hypothesis testing, the test statistic (e.g. *F*, *t*, *r*) with confidence intervals, effect sizes, degrees of freedom and *P* value noted<br>*Give P values as exact values whenever suitable.*
☒	☐	For Bayesian analysis, information on the choice of priors and Markov chain Monte Carlo settings
☒	☐	For hierarchical and complex designs, identification of the appropriate level for tests and full reporting of outcomes
☐	☒	Estimates of effect sizes (e.g. Cohen's *d*, Pearson's *r*), indicating how they were calculated

Our web collection on statistics for biologists contains articles on many of the points above.

Software and code

Policy information about availability of computer code

Data collection	No software was used for data collection
Data analysis	Code: https://github.com/scbe-lab/pristina-cell-type-atlas [DOI: 10.5281/zenodo.10671442].<br>EvidentialGene tr2aacds4.pl approach (March 2020 v4 version)<br>Transdecoder v5.5<br>diamond v2.0.8.146<br>EggNOG mapper v2<br>FastQC v0.11.9<br>CutAdapt v2.8<br>PairFQv0.17<br>SPLiTseq toolbox vl.0<br>Dropseq_tools v2.3.0<br>STAR v2.7.3a<br>Picard v2.21.1<br>Scanpy vl.9.3<br>Solo v0.l<br>Python v3.8.10<br>DESeq2 vl.30.0<br>R v4.0.3<br>OrthoFinder v2.3.8<br>BLAST+ v2.10.0

ComplexHeatmap v2.ll.l
ggplot2 v3.3.3
WGCNA v1.69
edgeR v3.32.1
limma v3.46.0
topGO v2.42.0
HCRProbeMaker v0.3.2
Fiji v2.9.0
bamtools v2.5.1
samtools v1.10

For manuscripts utilizing custom algorithms or software that are central to the research but not yet described in published literature, software must be made available to editors and reviewers. We strongly encourage code deposition in a community repository (e.g. GitHub). See the Nature Portfolio guidelines for submitting code & software for further information.

Data

Policy information about availability of data

All manuscripts must include a data availability statement. This statement should provide the following information, where applicable:

- Accession codes, unique identifiers, or web links for publicly available datasets
- A description of any restrictions on data availability
- For clinical datasets or third party data, please ensure that the statement adheres to our policy

The datasets supporting the conclusions of this article are available in:

Bioproject: PFUNA961657
GEO (scRNA-seq reads): GSE230505
BioSample (Iso-seq reads): SAMN34360745

BUSCO database https://busco.ezlab.org/
nr database https://www.ncbi.nlm.nih.gov/refseq/about/nonredundantproteins/
Pfam database http://pfam-legacy.xfam.org/
PANTHER database https://pantherdb.org/
SUPERFAMILY database https://supfam.org/SUPERFAMILY/
AnimalTFDB v3.0 https://guolab.wchscu.cn/AnimalTFDB
SwissProt https://www.uniprot.org/
EggNOG database http://eggnog5.embl.de
EpiFactors database https://epifactors.autosome.org/

Research involving human participants, their data, or biological material

Policy information about studies with human participants or human data. See also policy information about sex, gender (identity/presentation), and sexual orientation and race, ethnicity and racism.

Reporting on sex and gender	This study did not involve research in humans.
Reporting on race, ethnicity, or other socially relevant groupings	This study did not involve research in humans.
Population characteristics	This study did not involve research in humans.
Recruitment	This study did not involve research in humans.
Ethics oversight	This study did not involve research in humans.

Note that full information on the approval of the study protocol must also be provided in the manuscript.

Field-specific reporting

Please select the one below that is the best fit for your research. If you are not sure, read the appropriate sections before making your selection.

☒ Life sciences ☐ Behavioural & social sciences ☐ Ecological, evolutionary & environmental sciences

For a reference copy of the document with all sections, see nature.com/documents/nr-reporting-summary-flat.pdf

Life sciences study design

All studies must disclose on these points even when the disclosure is negative.

Sample size: No statistical method was used for determining sample size. The sample size for single cell experiments was calculated by performing pilot

Sample size	studies with different amounts of animals and evaluating the cell content using cell cytometry. The minimum required was determined in previous literature (Garcia-Castro et al Genome Biology 2021)
Data exclusions	We removed doublet detected by Solo and Scrublet from the dataset, eliminating 4,966 cells (6.1%).
Replication	Our dataset includes 3 biological replicates. All attempts at replication were successful.
Randomization	This study does not require randomization. Covariates (feeding conditions) are stated in the dataset. Our analysis does not study the effects of feeding but aims at generating a cell type atlas of wild type conditions. Therefore, this covariate is not relevant to our study.
Blinding	Blinding is not relevant for this study. The statistical analysis of our dataset does not have any variable sensitive to researcher blinding.

Reporting for specific materials, systems and methods

We require information from authors about some types of materials, experimental systems and methods used in many studies. Here, indicate whether each material, system or method listed is relevant to your study. If you are not sure if a list item applies to your research, read the appropriate section before selecting a response.

Materials & experimental systems

n/a	Involved in the study
☒	☐ Antibodies
☒	☐ Eukaryotic cell lines
☒	☐ Palaeontology and archaeology
☐	☒ Animals and other organisms
☒	☐ Clinical data
☒	☐ Dual use research of concern
☒	☐ Plants

Methods

n/a	Involved in the study
☒	☐ ChIP-seq
☒	☐ Flow cytometry
☒	☐ MRI-based neuroimaging

Animals and other research organisms

Policy information about studies involving animals; ARRIVE guidelines recommended for reporting animal research, and Sex and Gender in Research

Laboratory animals	Pristina leidyi worms were originally obtained from Carolina Biological Supply Company and established as a culture by Alexandra E. Bely around 2001 (doi:10.1242/dev.128.14.2781). Part of this culture was transfered and established at Oxford Brookes University in 2019.
Wild animals	This study did not involve wild animals.
Reporting on sex	Sex can not be determined, as the model organism does not reproduce sexually in lab conditions. Generally, annelids from this group are hermaphrodite when sexualised.
Field-collected samples	The study did not involve samples collected from the field.
Ethics oversight	No ethical approval is required to work with annelids.

Note that full information on the approval of the study protocol must also be provided in the manuscript.

Plants

Seed stocks	*Report on the source of all seed stocks or other plant material used. If applicable, state the seed stock centre and catalogue number. If plant specimens were collected from the field, describe the collection location, date and sampling procedures.*
Novel plant genotypes	*Describe the methods by which all novel plant genotypes were produced. This includes those generated by transgenic approaches, gene editing, chemical/radiation-based mutagenesis and hybridization. For transgenic lines, describe the transformation method, the number of independent lines analyzed and the generation upon which experiments were performed. For gene-edited lines, describe the editor used, the endogenous sequence targeted for editing, the targeting guide RNA sequence (if applicable) and how the editor was applied.*
Authentication	*Describe any authentication procedures for each seed stock used or novel genotype generated. Describe any experiments used to assess the effect of a mutation and, where applicable, how potential secondary effects (e.g. second site T-DNA insertions, mosiacism, off-target gene editing) were examined.*
